# Causal Relationship between Obesity and Vitamin D Status: Bi-Directional Mendelian Randomization Analysis of Multiple Cohorts

**DOI:** 10.1371/journal.pmed.1001383

**Published:** 2013-02-05

**Authors:** Karani S. Vimaleswaran, Diane J. Berry, Chen Lu, Emmi Tikkanen, Stefan Pilz, Linda T. Hiraki, Jason D. Cooper, Zari Dastani, Rui Li, Denise K. Houston, Andrew R. Wood, Karl Michaëlsson, Liesbeth Vandenput, Lina Zgaga, Laura M. Yerges-Armstrong, Mark I. McCarthy, Josée Dupuis, Marika Kaakinen, Marcus E. Kleber, Karen Jameson, Nigel Arden, Olli Raitakari, Jorma Viikari, Kurt K. Lohman, Luigi Ferrucci, Håkan Melhus, Erik Ingelsson, Liisa Byberg, Lars Lind, Mattias Lorentzon, Veikko Salomaa, Harry Campbell, Malcolm Dunlop, Braxton D. Mitchell, Karl-Heinz Herzig, Anneli Pouta, Anna-Liisa Hartikainen, the Genetic Investigation of Anthropometric Traits (GIANT) consortium, Elizabeth A. Streeten, Evropi Theodoratou, Antti Jula, Nicholas J. Wareham, Claes Ohlsson, Timothy M. Frayling, Stephen B. Kritchevsky, Timothy D. Spector, J. Brent Richards, Terho Lehtimäki, Willem H. Ouwehand, Peter Kraft, Cyrus Cooper, Winfried März, Chris Power, Ruth J. F. Loos, Thomas J. Wang, Marjo-Riitta Järvelin, John C. Whittaker, Aroon D. Hingorani, Elina Hyppönen

**Affiliations:** 1 Centre for Paediatric Epidemiology and Biostatistics and MRC Centre of Epidemiology for Child Health, UCL Institute of Child Health, London, United Kingdom; 2 Department of Biostatistics, Boston University School of Public Health, Boston, Massachusetts, United States of America; 3 Institute for Molecular Medicine Finland FIMM, University of Helsinki, Helsinki, Finland; 4 National Institute for Health and Welfare, Helsinki, Finland; 5 Department of Internal Medicine, Division of Endocrinology and Metabolism, Medical University of Graz, Austria; 6 Department of Epidemiology and Biostatistics, EMGO Institute for Health and Care Research, VU University Medical Centre, Amsterdam, The Netherlands; 7 Program in Molecular and Genetic Epidemiology, Harvard School of Public Health, Boston, Massachusetts, United States of America; 8 Juvenile Diabetes Research Foundation/Wellcome Trust Diabetes and Inflammation Laboratory, Department of Medical Genetics, Cambridge Institute for Medical Research, University of Cambridge, Cambridge, United Kingdom; 9 Department of Epidemiology, Biostatistics and Occupational Health, Lady Davis Institute, Jewish General Hospital, McGill University, Montreal, Quebec, Canada; 10 Departments of Medicine, Human Genetics, Epidemiology and Biostatistics, Lady Davis Institute, Jewish General Hospital, McGill University, Montreal, Quebec, Canada; 11 Department of Internal Medicine, Section on Gerontology and Geriatric Medicine, Wake Forest School of Medicine, Winston Salem, North Carolina, United States of America; 12 Genetics of Complex Traits, Peninsula College of Medicine and Dentistry, University of Exeter, Exeter, United Kingdom; 13 Department of Surgical Sciences, Uppsala University, Uppsala, Sweden; 14 Center for Bone and Arthritis Research, Department of Internal Medicine, Institute of Medicine, University of Gothenburg, Gothenburg, Sweden; 15 Centre for Population Health Sciences, University of Edinburgh, Edinburgh, United Kingdom; 16 Andrija Stampar School of Public Health, Medical School University of Zagreb, Zagreb, Croatia; 17 University of Maryland School of Medicine, Division of Endocrinology, Baltimore, Maryland, United States of America; 18 Oxford Centre for Diabetes, Endocrinology and Metabolism, University of Oxford, Churchill Hospital, Headington, Oxford, United Kingdom; 19 Wellcome Trust Centre for Human Genetics, University of Oxford, Oxford, United Kingdom; 20 Oxford NIHR Biomedical Research Centre, Churchill Hospital, Headington, Oxford, United Kingdom; 21 National Heart, Lung, and Blood Institute's Framingham Heart Study, Framingham, Massachusetts, United States of America; 22 Institute of Health Sciences and Biocenter Oulu, University of Oulu, Oulu, Finland; 23 LURIC Study non-profit LLC, Freiburg, Germany and Mannheim Institute of Public Health, Social and Preventive Medicine, Mannheim Medical Faculty, University of Heidelberg, Mannheim, Germany; 24 MRC Lifecourse Epidemiology Unit, University of Southampton, Southampton, United Kingdom; 25 NIHR Musculoskeletal BRU, Botnar Research Centre, Oxford, United Kingdom; 26 MRC Lifecourse Epidemiology Unit, University of Southampton, Southampton, United Kingdom; 27 Research Centre of Applied and Preventive Cardiovascular Medicine, University of Turku and Department of Clinical Physiology and Nuclear Medicine, University of Turku and Turku University Hospital, Turku, Finland; 28 Department of Medicine, University of Turku and Turku University Hospital, Turku, Finland; 29 Department of Biostatistical Sciences, Division of Public Health Sciences, Wake Forest School of Medicine, Winston Salem, North Carolina, United States of America; 30 Clinical Research Branch, Harbor Hospital, Baltimore, Maryland, United States of America; 31 Department of Medical Sciences, Uppsala University, Uppsala, Sweden; 32 Department of Medical Epidemiology and Biostatistics, Karolinska Institutet, Stockholm, Sweden; 33 Colon Cancer Genetics Group and Academic Coloproctology, Institute of Genetics and Molecular Medicine, University of Edinburgh, United Kingdom; 34 MRC Human Genetics Unit Western General Hospital Edinburgh, United Kingdom; 35 Institute of Biomedicine, University of Oulu, Oulu, Finland; 36 Department of Psychiatry, Kuopio University Hospital, Kuopio, Finland; 37 Department of Public Health Science and General Practice, University of Oulu, Oulu, Finland; 38 Department of Obstetrics and Gynaecology and Public Health and General Practice, University of Oulu, Oulu, Finland; 39 MRC Epidemiology Unit, Institute of Metabolic Science, Addenbrooke's Hospital, Cambridge, United Kingdom; 40 Department of Twin Research and Genetic Epidemiology, King's College London, London, United Kingdom; 41 Department of Clinical Chemistry, Fimlab Laboratories, Tampere University Hospital and University of Tampere, Tampere, Finland; 42 Department of Haematology, University of Cambridge, United Kingdom; 43 Wellcome Trust Sanger Institute, Hinxton, Cambridge, United Kingdom; 44 NHS Blood and Transplant, Cambridge, United Kingdom; 45 Synlab Academy, Mannheim, Germany; 46 Mannheim Institute of Public Health, Social and Preventive Medicine, Mannheim Medical Faculty, University of Heidelberg, Mannheim, Germany; 47 Cardiology Division, Massachusetts General Hospital, Boston, Massachusetts, United States of America; 48 Department of Biostatistics and Epidemiology, School of Public Health, MRC-HPA Centre for Environment and Health, Imperial College, Faculty of Medicine, London, United Kingdom; 49 Department of Children, Young People and Families, National Institute for Health and Welfare, Oulu, Finland; 50 Department of Epidemiology and Population Health, London School of Hygiene and Tropical Medicine, London, United Kingdom; 51 Quantitative Sciences, GlaxoSmithKline, Stevenage, United Kingdom; 52 Genetic Epidemiology Group, Department of Epidemiology and Public Health, Division of Population Health, University College London, London, United Kingdom; 53 Division of Medicine, Centre for Clinical Pharmacology, University College London, London, United Kingdom; Centre for Biomedicine, EURAC, Italy

## Abstract

**Background:**

Obesity is associated with vitamin D deficiency, and both are areas of active public health concern. We explored the causality and direction of the relationship between body mass index (BMI) and 25-hydroxyvitamin D [25(OH)D] using genetic markers as instrumental variables (IVs) in bi-directional Mendelian randomization (MR) analysis.

**Methods and Findings:**

We used information from 21 adult cohorts (up to 42,024 participants) with 12 BMI-related SNPs (combined in an allelic score) to produce an instrument for BMI and four SNPs associated with 25(OH)D (combined in two allelic scores, separately for genes encoding its synthesis or metabolism) as an instrument for vitamin D. Regression estimates for the IVs (allele scores) were generated within-study and pooled by meta-analysis to generate summary effects.

Associations between vitamin D scores and BMI were confirmed in the Genetic Investigation of Anthropometric Traits (GIANT) consortium (*n* = 123,864). Each 1 kg/m^2^ higher BMI was associated with 1.15% lower 25(OH)D (*p = *6.52×10^−27^). The BMI allele score was associated both with BMI (*p = *6.30×10^−62^) and 25(OH)D (−0.06% [95% CI −0.10 to −0.02], *p* = 0.004) in the cohorts that underwent meta-analysis. The two vitamin D allele scores were strongly associated with 25(OH)D (*p*≤8.07×10^−57^ for both scores) but not with BMI (synthesis score, *p* = 0.88; metabolism score, *p* = 0.08) in the meta-analysis. A 10% higher genetically instrumented BMI was associated with 4.2% lower 25(OH)D concentrations (IV ratio: −4.2 [95% CI −7.1 to −1.3], *p* = 0.005). No association was seen for genetically instrumented 25(OH)D with BMI, a finding that was confirmed using data from the GIANT consortium (*p*≥0.57 for both vitamin D scores).

**Conclusions:**

On the basis of a bi-directional genetic approach that limits confounding, our study suggests that a higher BMI leads to lower 25(OH)D, while any effects of lower 25(OH)D increasing BMI are likely to be small. Population level interventions to reduce BMI are expected to decrease the prevalence of vitamin D deficiency.

*Please see later in the article for the Editors' Summary*

## Abstract 2

A mendelian randomization study based on data from multiple cohorts conducted by Karani Santhanakrishnan Vimaleswaran and colleagues re-examines the causal nature of the relationship between vitamin D levels and obesity.

## Editors' Summary

**Background:**

Obesity—having an unhealthy amount of body fat—is increasing worldwide. In the US, for example, a third of the adult population is now obese. Obesity is defined as having a body mass index (BMI, an indicator of body fat calculated by dividing a person's weight in kilograms by their height in meters squared) of more than 30.0 kg/m^2^. Although there is a genetic contribution to obesity, people generally become obese by consuming food and drink that contains more energy than they need for their daily activities. Thus, obesity can be prevented by having a healthy diet and exercising regularly. Compared to people with a healthy weight, obese individuals have an increased risk of developing diabetes, heart disease and stroke, and tend to die younger. They also have a higher risk of vitamin D deficiency, another increasingly common public health concern. Vitamin D, which is essential for healthy bones as well as other functions, is made in the skin after exposure to sunlight but can also be obtained through the diet and through supplements.

**Why Was This Study Done?:**

Observational studies cannot prove that obesity causes vitamin D deficiency because obese individuals may share other characteristics that reduce their circulating 25-hydroxy vitamin D [25(OH)D] levels (referred to as confounding). Moreover, observational studies cannot indicate whether the larger vitamin D storage capacity of obese individuals (vitamin D is stored in fatty tissues) lowers their 25(OH)D levels or whether 25(OH)D levels influence fat accumulation (reverse causation). If obesity causes vitamin D deficiency, monitoring and treating vitamin D deficiency might alleviate some of the adverse health effects of obesity. Conversely, if low vitamin D levels cause obesity, encouraging people to take vitamin D supplements might help to control the obesity epidemic. Here, the researchers use bi-directional “Mendelian randomization” to examine the direction and causality of the relationship between BMI and 25(OH)D. In Mendelian randomization, causality is inferred from associations between genetic variants that mimic the influence of a modifiable environmental exposure and the outcome of interest. Because gene variants do not change over time and are inherited randomly, they are not prone to confounding and are free from reverse causation. Thus, if a lower vitamin D status leads to obesity, genetic variants associated with lower 25(OH)D concentrations should be associated with higher BMI, and if obesity leads to a lower vitamin D status, then genetic variants associated with higher BMI should be associated with lower 25(OH)D concentrations.

**What Did the Researchers Do and Find?:**

The researchers created a “BMI allele score” based on 12 BMI-related gene variants and two “25(OH)D allele scores,” which are based on gene variants that affect either 25(OH)D synthesis or breakdown. Using information on up to 42,024 participants from 21 studies, the researchers showed that the BMI allele score was associated with both BMI and with 25(OH)D levels among the study participants. Based on this information, they calculated that each 10% increase in BMI will lead to a 4.2% decrease in 25(OH)D concentrations. By contrast, although both 25(OH)D allele scores were strongly associated with 25(OH)D levels, neither score was associated with BMI. This lack of an association between 25(OH)D allele scores and obesity was confirmed using data from more than 100,000 individuals involved in 46 studies that has been collected by the GIANT (Genetic Investigation of Anthropometric Traits) consortium.

**What Do These Findings Mean?:**

These findings suggest that a higher BMI leads to a lower vitamin D status whereas any effects of low vitamin D status on BMI are likely to be small. That is, these findings provide evidence for obesity as a causal factor in the development of vitamin D deficiency but not for vitamin D deficiency as a causal factor in the development of obesity. These findings suggest that population-level interventions to reduce obesity should lead to a reduction in the prevalence of vitamin D deficiency and highlight the importance of monitoring and treating vitamin D deficiency as a means of alleviating the adverse influences of obesity on health.

**Additional Information:**

Please access these Web sites via the online version of this summary at http://dx.doi.org/10.1371/journal.pmed.1001383.

- The US Centers for Disease Control and Prevention provides information on all aspects of overweight and obesity (in English and Spanish); a data brief provides information about the vitamin D status of the US population
- The World Health Organization provides information on obesity (in several languages)
- The UK National Health Service Choices website provides detailed information about obesity and a link to a personal story about losing weight; it also provides information about vitamin D
- The International Obesity Taskforce provides information about the global obesity epidemic
- The US Department of Agriculture's ChooseMyPlate.gov website provides a personal healthy eating plan; the Weight-control Information Network is an information service provided for the general public and health professionals by the US National Institute of Diabetes and Digestive and Kidney Diseases (in English and Spanish)
- The US Office of Dietary Supplements provides information about vitamin D (in English and Spanish)
- MedlinePlus has links to further information about obesity and about vitamin D (in English and Spanish)
- Wikipedia has a page on Mendelian randomization (note: Wikipedia is a free online encyclopedia that anyone can edit; available in several languages)
- Overview and details of the collaborative large-scale genetic association study (D-CarDia) provide information about vitamin D and the risk of cardiovascular disease, diabetes and related traits

## Introduction

The prevalence of obesity has increased in the last two decades and it is presently the most common and costly nutritional problem [1]–[4]. In the United States, one-third of the population is affected by obesity, according to the National Health and Nutrition Examination Survey [5]. Despite a known genetic contribution, the increase in obesity prevalence has been largely attributed to lifestyle changes, which means that it is amenable to modification through public health and other interventions [6].

Vitamin D deficiency is another increasingly prevalent public health concern in developed countries [7]–[9], and there is evidence that vitamin D metabolism, storage, and action both influence and are influenced by adiposity. Observational studies have reported an increased risk of vitamin D deficiency in those who are obese; however, the underlying explanations and direction of causality are unclear [10]. Active vitamin D (1,25-dihydroxyvitamin D) may influence the mobilisation of free fatty acids from the adipose tissue [11]. In vitro experiments in rats have also shown that large doses of vitamin D_2_ lead to increases in energy expenditure due to uncoupling of oxidative phosphorylation in adipose tissues [12]. However, randomized controlled trials (RCTs) testing the effect of vitamin D supplementation on weight loss in obese or overweight individuals have provided inconsistent findings [13]–[15]. It has also been suggested that obesity could result from an excessive adaptive winter response, and that the decline in vitamin D skin synthesis due to reduced sunlight exposure contributes to the tendency to increase fat mass during the colder periods of the year [16],[17]. However, vitamin D is stored in the adipose tissue and, hence, perhaps the most likely explanation for the association is that the larger storage capacity for vitamin D in obese individuals leads to lower circulating 25-hydroxyvitamin D [25(OH)D] concentrations, a marker for nutritional status [18].

In the Mendelian randomization (MR) approach, causality is inferred from associations between genetic variants that mimic the influence of a modifiable environmental exposure and the outcome of interest [19]. If lower vitamin D intake/status is causally related to obesity, a genetic variant associated with lower 25(OH)D concentrations should be associated with higher body mass index (BMI) (in proportion to the effect on 25(OH)D). Conversely, if obesity leads to lower vitamin D status, then genetic variants associated with higher BMI should be related to lower 25(OH)D concentrations. The genetic associations, unlike the directly observed associations for vitamin D intake/status, should be less prone to confounding by lifestyle and socio-economic factors and be free from reverse causation as genotypes are invariant and assigned at random before conception [20]. The use of multiple SNPs to index the intermediate exposure of interest increases power and reduces the risk of alternative biological pathways (pleiotropy) affecting the observed associations between the genotype and the outcome [21],[22].

In the present study, we investigated the relationship between BMI, a commonly used measure for monitoring the prevalence of obesity at the population level, and vitamin D status and we inferred causality by using genetic variants as instruments in bi-directional MR analyses. Meta-analysis included data from 21 studies comprising up to 42,024 individuals.

## Methods

### Ethics Statement

All participants provided written, informed consent, and ethical permission was granted by the local research ethics committees for all participating studies.

### Participants

The collaboration investigating the association of vitamin D and the risk of cardiovascular disease and related traits (D-CarDia) consists of European ancestry cohorts from the United Kingdom (UK), United States (US), Canada, Finland, Germany, and Sweden. This study comprised a meta-analysis of directly genotyped and imputed SNPs from 21 cohorts totalling 42,024 individuals (Table 1). An expanded description of the participating studies is provided in the Text S2.

**Table 1:** Characteristics of the study cohorts stratified by sex.

Study Name	Sample Size^a^, *n* (Men/Women)	Men			Women			Combined		
		Age (y) (Mean ± SD)	Geometric Mean		Age (y) (Mean ± SD)	Geometric Mean		Age (y) (Mean ± SD)	Geometric Mean	
			BMI (kg/m^2^)	25(OH)D (nmol/l)		BMI (kg/m^2^)	25(OH)D (nmol/l)		BMI (kg/m^2^)	25(OH)D (nmol/l)
1958 British Birth cohort (1958BC)	3,711/3,703	45.2±0.4	27.4	53.1	45.2±0.4	26.3	51.2	45.2±0.4	26.9	52.1
1966 North Finland Birth cohort (NFBC1966)	2,192/2,261	31.1±0.40	25.0	63.8	31.1±0.3	23.7	62.7	31.1±0.4	24.3	63.2
Framingham Heart Study (FHS)	2,678/2,978	46.9±13.0	25.3	68.4	46.4±13.1	25.8	71.9	46.6±13.1	26.8	69.7
The Ludwigshafen Risk and Cardiovascular Health study (LURIC)	2,299/999	61.8±10.7	27.3	39.5	64.7±10.2	26.9	32.6	62.6±10.6	27.2	37.3
Hertfordshire Cohort Study (HCS)	586/624	64.3±2.6	26.7	44.8	65.7±2.5	26.9	39.0	65.0±2.6	26.9	41.7
UK Blood Services Common Control Collection (UKBS-CC)	1,310/1,298	45.2±11.8	26.3	50.4	42.3±12.5	25.5	55.2	43.8±12.2	26.1	52.5
Young Finns	907/1,077	37.6±5.1	26.6	54.1	37.6±5.0	24.8	57.9	37.6±5.0	25.5	56.3
Canadian Multicentre osteoporosis Study (CaMos)	709/1,588	62.3±18.2	26.8	64.1	65.2±15.8	26.8	64.1	64.3±16.6	26.8	64.1
Twins UK	176/1,754	51.0±13.2	26.1	64.1	51.2±12.9	25.0	68.7	51.2±13.2	25.3	68.0
Health, Aging and Body Composition study (Health ABC)	829/729	74.9±2.9	26.8	68.4	74.7±2.8	25.5	66.3	74.8±2.9	26.3	67.7
The Health Professionals Follow-up Study-CHD (HPFS-CHD)	1,245/−	63.8±8.6	25.5	56.8	—	—	—	—	—	—
InCHIANTI Study	496/598	67.2±15.4	26.8	50.4	69.1±15.6	26.8	38.1	68.3±15.5	26.8	43.4
Uppsala Longitudinal Study of Adult Men (ULSAM)	1,194/−	71.0±0.6	26.1	65.8	—	—	—	—	—	—
Prospective Investigation of the Vasculature in Uppsala Seniors (PIVUS)	500/499	70.1±0.2	26.8	56.8	70.2±0.1	26.7	51.9	70.2±0.2	26.7	54.3
The Gothenburg Osteoporosis and Obesity Determinants study (GOOD)	921/−	18.9±0.6	22.1	61.8	—	—	—	—	—	—
Cancer Genetic Markers of Susceptibility, case control study of breast cancer (NHS-CGEMS)	−/870	—	—	—	59.6±5.8	25.0	74.4	—	—	—
Health2000 GenMets Study (GENMETS)	397/424	49.2±10.4	25.3	44.7	52.0±11.6	24.8	45.2	50.7±11.1	25.0	44.7
MRC Ely study	323/435	53.8±7.8	25.8	57.7	53.2±7.6	25.3	50.1	53.5±7.7	25.5	53.2
Study of Colorectal Cancer in Scotland (SOCCS)	336/328	51.5±5.9	26.8	33.3	50.9±5.9	26.6	34.9	51.2±5.1	26.8	34.3
Nurses' Health Study- Case-control study of type II diabetes (NHS-T2D)	−/720	—	—	—	56.5±6.9	27.1	53.5	—	—	—
Amish Family Osteoporosis Study (AFOS)	141/189	48.5±13.9	25.8	54.3	49.4±13.8	27.7	53.2	49.0±13.9	26.8	53.8

a Sample size based on available information on body mass index and 25(OH)D.

To replicate our findings on the association between the vitamin D-related SNPs and allele scores with BMI, we used the data from the genome-wide meta-analyses on BMI conducted as part of the Genetic Investigation of Anthropometric Traits (GIANT) consortium [23]. The GIANT meta-analyses consisted of 46 studies with up to 123,865 adults of European ancestry, including the 1958 British Birth Cohort, Framingham Heart study, Nurses' Health Study, Twins UK, UK Blood Services Common Control Collection, the Amish Family Osteoporosis Study, Health2000 GENMETS sub-sample, and Northern Finland Birth Cohort 1966, which were also part of the D-CarDia collaboration.

### Genotyping

We selected 12 established BMI-related SNPs (fat mass and obesity-associated, [*FTO*]- rs9939609, melanocortin 4 receptor [*MC4R*]- rs17782313, transmembrane protein 18 [*TMEM18*]- rs2867125, SH2B adaptor protein 1 [*SH2B1*]- rs7498665, brain-derived neurotrophic factor [*BDNF*]- rs4074134, potassium channel tetramerisation domain containing 15 [*KCTD15*]- rs29941, ets variant 5 [*ETV5*]- rs7647305, SEC16 homolog B [*SEC16B*]- rs10913469, Fas apoptotic inhibitory molecule 2 [*FAIM2*]- rs7138803, neuronal growth regulator 1 [*NEGR1*]- rs3101336, mitochondrial carrier 2 [*MTCH2*]- rs10838738, and glucosamine-6-phosphate deaminase 2 [*GNPDA2*]- rs10938397) for our analysis based on the study by Li et al. [24] and previously published genome-wide association studies for obesity-related traits [23],[25],[26]. The four vitamin D-related SNPs (*DHCR7*- rs12785878, *CYP2R1*- rs10741657, *GC*- rs2282679, and *CYP24A1*- rs6013897) were chosen on the basis of the recent genome-wide association study on 25(OH)D [27]. The studies that did not have genotyped data analysed imputed or proxy SNPs (r^2^ = 1) as available (with a call threshold of 0.9 for the SNPs imputed with Impute; for those imputed with MACH, a call threshold of 0.8 was used) [28]. The genetic data for most studies were obtained from genome-wide association platforms, but for some studies, variants were genotyped de novo (MRC Ely, the Canadian Multicentre Osteoporosis Study, the Hertfordshire cohort study) or obtained through metabochip custom array (MRC Ely). Five studies did not have all the BMI-related SNPs (Framingham Heart Study [one missing SNP], Hertfordshire cohort study [three missing SNPs], InCHIANTI [two missing SNPs], PIVUS [two missing SNPs], and ULSAM [three missing SNPs]) and were still included in the BMI allele score analysis. Table S1 shows the minor allele frequencies for the BMI and vitamin D SNPs that were included in the analysis. A detailed description of the genotyping methods is provided in Text S2.

### Statistical Analysis

Analyses in each study were performed according to a standardized analysis plan. When used as outcome variables, 25(OH)D and BMI were natural log transformed to be more closely approximated by normal distributions. If multiplied by 100, coefficients from linear regression models with ln transformed outcomes can be interpreted as the percentage difference in the outcome [29]. Models with BMI as an outcome were adjusted for age, sex, geographical site, and/or principal components from population stratification analysis (depending on data available); models with 25(OH)D as the outcome were additionally adjusted for month of blood sample collection (as a categorical variable) to account for seasonal variation and laboratory batch, where relevant. To assess the BMI relationship with 25(OH)D and vice versa, each study ran linear regression models adjusting for the covariates listed for each outcome, and the models were repeated stratifying by sex.

For the BMI SNPs, the effect allele was the BMI raising allele as established by Speliotes et al. [23]. We created a weighted score in each study [30], by multiplying each SNP (coded as 0–2) by a weight based on its effect size with BMI in the meta-analysis by Speliotes et al. [23]. The weighted BMI allele score was rescaled over the sum of weights for the available SNPs in each study to facilitate interpretation [30]. For the vitamin D SNPs, the effect allele was the 25(OH)D lowering allele as established by the SUNLIGHT Consortium [27]. As external weights were not available and the use of internal weights could bias the instrumental variable (IV) results [31], we performed an unweighted allele score analysis for the vitamin D SNPs. Vitamin D SNPs were used to form two separate allele scores [32]: a “synthesis” allele score, created by summing the risk alleles in *DHCR7* and *CYP2R1*, and a “metabolism” allele score, created by summing the risk alleles in *GC* and *CYP24A1* (Figure S1). Synthesis allele score was not created for the LURIC study (one missing SNP) and both synthesis and metabolism allele scores were not created for the MRC Ely study (two missing SNPs). The synthesis allele score included the SNPs that contribute directly to the production of 25(OH)D, and hence, for which the association with the outcome can be readily estimated based on the magnitude of the association between the score and 25(OH)D [32]. All analyses were done separately for the “metabolism” SNPs that are involved in the clearance or transport of 25(OH)D (with possible influences on bioavailability [33]) as the quantification of the association with the outcome based on the observed SNP-25(OH)D association is more difficult [32]. We also evaluated the joint contribution of synthesis and metabolism scores on BMI by including both vitamin D scores as separate variables in a multiple regression model. To examine the strength of the allele scores as instruments, the F-statistic was approximated from the proportion of variation in the respective phenotype (R^2^) explained by the allele score, [F-stat = (R^2^×(*n*−2))/(1−R^2^)] [34].

To confirm our findings on the association between the vitamin D-related SNPs and allele scores with BMI in a larger sample, we used the summary statistics for the four vitamin D-related SNPs from the GIANT consortium. These SNPs were combined into synthesis and metabolism allele scores using an approximation method as previously described [35]. The individual SNP association with BMI is then weighted according to its predefined effect size and meta-analysed using the inverse-variance method with the other SNPs in the score [35]. The formal MR analyses to estimate the possible causal effect of BMI on 25(OH)D (and vice versa) were done using the IV ratio method [20],[36]. To estimate the IV ratio for the BMI effect on 25(OH)D, the meta-analysed association of the BMI allele score with 25(OH)D was divided by the association of BMI allele score with BMI. The variance for the IV ratio was estimated using a Taylor expansion [36]. The corresponding calculation was done to establish the 25(OH)D effect on BMI, with the IV ratio method applied separately for the two vitamin D allele scores. The joint contribution of the two vitamin D scores on BMI was assessed by multivariate meta-analysis [37], which incorporated the covariance matrix as estimated by study specific analyses.

In the presence of heterogeneity of association between the studies, random effects meta-analyses [38] were run, otherwise fixed effects models were used. Univariate meta-regression models were run to assess differences in the observed associations by study level factors of sex, average BMI (BMI≤25 kg/m^2^ versus >25 kg/m^2^), the average age of participants (≤40, 41–60, and ≥61 y old), continent (North America versus Europe), and vitamin D assay (radio-immunoassay, enzyme-linked radio-immunoassay, and mass spectrometry). Power calculations for IV regression were performed by simulation [32] on the basis of associations observed between the phenotypes and their genetic proxies. For comparability across instruments/outcomes, power was determined for 0.02 log unit increase/decrease by decile, approximately corresponding to the association observed between BMI and 25(OH)D. To evaluate the ability to detect weaker effects on BMI using the synthesis and metabolism scores, power was also calculated for a 50% weaker effect (0.01 log unit increase/decrease). All meta-analyses and power calculations were performed at the Institute of Child Health (University College London, London) using STATA version 12 [39].

## Results

### Phenotypic Association between BMI and 25(OH)D Concentrations

**Figure 1:**
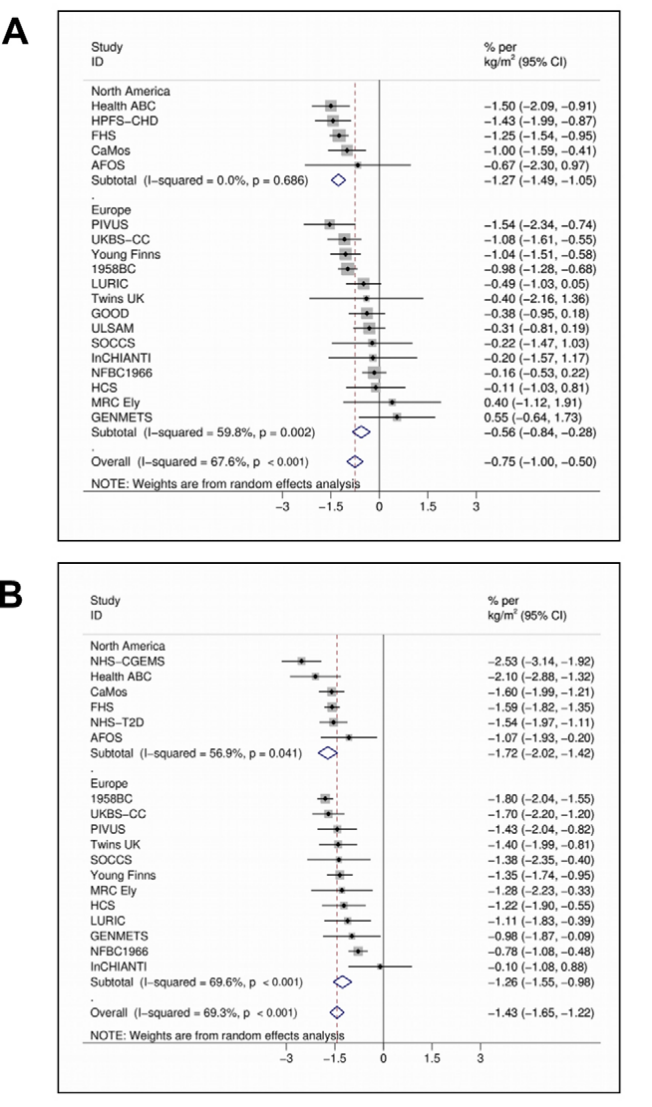
Random effects meta-analysis of the BMI association with 25(OH)D in men (A) (*n* = 20,950) and women (B) (*n* = 21,074). 95% confidence intervals given by error bars.

In the meta-analyses of 21 studies, each unit (kg/m^2^) increase in BMI was associated with 1.15% (95% CI 0.94%–1.36%, *p* = 6.52×10^−27^) lower concentrations of 25(OH)D after adjusting for age, sex, laboratory batch, month of measurement, and principal components. The inverse association between BMI and 25(OH)D was stronger among the studies from North America than those from Europe (−1.58% [−1.81% to −1.36%], *p* = 1.01×10^−43^ versus −0.91% [−1.18% to −0.64%], *p* = 4.55×10^−11^; *p_meta-regression_* = 0.004) and for women than men (−1.43% [−1.65% to −1.22%], *p* = 1.13×10^−38^ versus −0.75% [−1.00% to −0.50%], *p* = 3.89×10^−9^; *p_meta-regression_* = 4.10×10^−4^) while no variation was seen by average age (*p_meta-regression_* = 0.78) or BMI (*p_meta-regression_* = 0.48) (Figure 1A and 1B).

### Evaluation of Causal Association Using MR Approach

**Figure 2:**
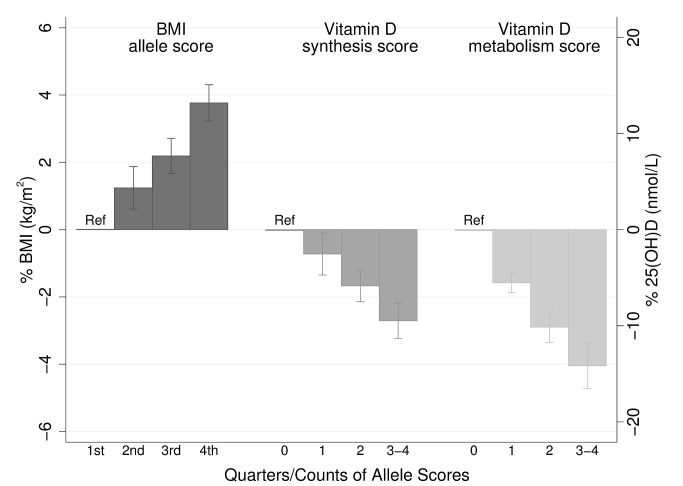
Meta-analysis of the BMI allele score association with BMI (*n* = 32,391), and the vitamin D synthesis (*n* = 35,873) and metabolism (*n* = 38,191) allele score association with 25(OH)D. 95% confidence intervals given by error bars.

**Figure 3:**
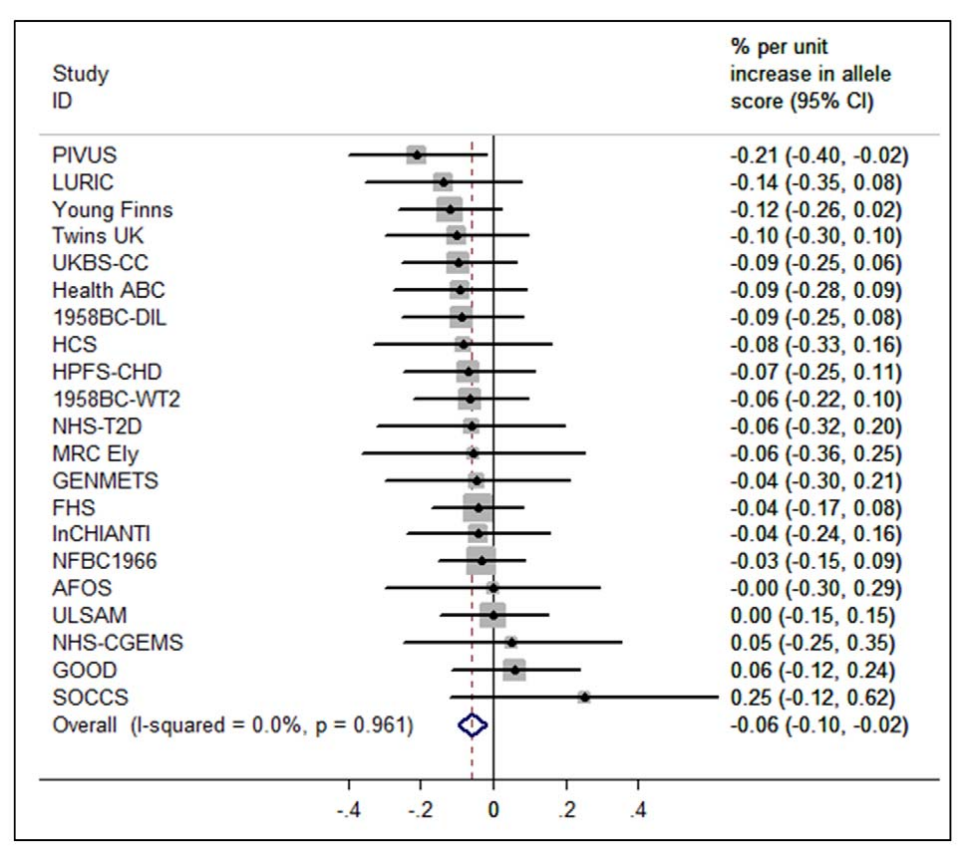
Meta-analysis of the BMI allele score association with 25(OH)D (*n* = 31,120). 95% confidence intervals given by error bars.

**Figure 4:**
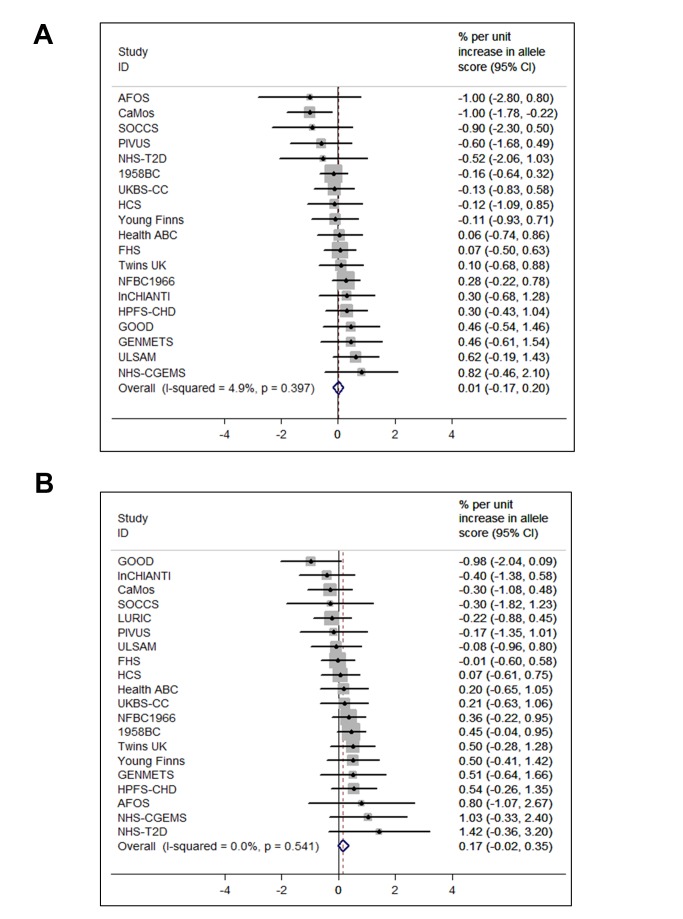
Meta-analysis of the synthesis allele score association with BMI (A) (*n* = 36,553) and the metabolism allele score association with BMI (B) (*n* = 40,367). 95% confidence intervals given by error bars.

The BMI allele score created from the 12 BMI-related SNPs showed a positive dose-response association with BMI (per unit increase 0.14% [0.12%–0.16%], *p* = 6.30×10^−62^), and both vitamin D allele scores showed the expected strong associations with 25(OH)D (per allele in synthesis score: −3.47% [−3.90% to −3.05%], *p* = 8.07×10^−57^; metabolism allele score: −5.38% [−5.84% to −4.93%], *p* = 1.07×10^−118^) (Figures 2, S2, and S3). The BMI allele score was also associated with 25(OH)D concentrations (per unit increase −0.06%, [−0.10% to −0.02%], *p* = 0.004) (Figure 3), while no association with BMI was seen for either the vitamin D synthesis or metabolism allele scores (per allele in synthesis score: 0.01% [−0.17% to 0.20%], *p* = 0.88, metabolism allele score: 0.17% [−0.02% to 0.35%], *p* = 0.08]) (Figure 4A and 4B). Analyses of joint effects by synthesis and metabolism scores provided no evidence for an association between 25(OH)D and BMI (per allele in synthesis score −0.03% [−0.23% to 0.16%] and metabolism score 0.17% [−0.04% to 0.37%], joint contribution *p* = 0.26).

In the analyses to establish the direction and causality of BMI–25(OH)D association by the use of the IV ratio, BMI was associated with 25(OH)D: each 10% increase in BMI lead to a 4.2% decrease in 25(OH)D concentrations (−7.1% to −1.3%; *p* = 0.005). However, the IV ratio analyses provided little evidence for a causal effect of 25(OH)D on BMI (*p*≥0.08 for both). We have summarised the coefficients for the MR analyses in Table 2.

**Table 2:** Summary of the coefficients used for IV ratio analyses.

IV	Allele Score with the Intermediate Trait	Allele Score with the Outcome	IV Ratio^a^	
	Coefficient, % (95% CI)	Coefficient,% (95% CI)	Coefficient (95% CI)	*p*-Value
**BMI risk score**	0.14 (0.12–0.16)	−0.06 (−0.1 to −0.02)	−0.42 (−0.71 to −0.13)	0.005
**Synthesis score**	−3.47 (−3.90 to −3.05)	0.01 (−0.17 to 0.20)	−0.00 (−0.06 to 0.05)	0.88
**Metabolism score**	−5.38 (−5.84 to −4.93)	0.17 (−0.02 to 0.35)	−0.03 (−0.06 to 0.01)	0.08

a Calculated as the ratio between the allele score association with the outcome and intermediate trait. Coefficients can be interpreted as percent change in the outcome by percent change in the intermediate trait.

The lack of association of the vitamin D allele scores with BMI was further confirmed using the GIANT consortium including 123,864 individuals in 46 studies [23]: neither the synthesis nor the metabolism allele score showed any evidence for an association with BMI (*p*≥0.57 for both) (Table 3).

**Table 3:** Results for the association between vitamin D SNPs/allele scores and BMI from the GIANT consortium.

SNPs/Allele Scores	Gene Symbol	Per Allele Change in BMI, kg/m^2^ (95% CI)	*p*-Value
rs12785878	*DHCR7*	0.001 (−0.01 to 0.009)	0.78
rs10741657	*CYP2R1*	−0.005 (−0.004 to 0.01)	0.30
Synthesis allele score (rs12785878+rs10741657)	*DHCR7* + *CYP2R1*	−0.002 (−0.009 to 0.005)^a^	0.57
rs2282679	*GC*	0.001 (−0.011 to 0.010)	0.91
rs6013897	*CYP24A1*	0.003 (−0.008 to 0.014)	0.61
Metabolism allele score (rs2282679+rs6013897)	*GC* + *CYP24A1*	0.002 (−0.006 to 0.009)^a^	0.67

The GIANT meta-analyses consisted of 46 studies with up to 123,865 adults of European ancestry [23], including the 1958 British Birth Cohort, Framingham Heart study, Nurses' Health Study, Twins UK, UK Blood Services Common Control Collection, the Amish Family Osteoporosis Study, Health2000 GENMETS sub-sample, and Northern Finland Birth Cohort 1966, which were also part of the D-CarDia collaboration.

a Calculated as described in Ehret et al. [35].

### Additional Analyses

#### Validation of the genetic instruments

The BMI SNPs and the vitamin D SNPs were all individually associated with BMI and 25(OH)D, respectively (Figures S4 and S5). The exception was *KCTD15* SNP, which despite previous evidence for an association [25], was not associated with BMI in our meta-analyses. Across the studies, the 12 BMI SNPs combined as the BMI allele score explained 0.97% of the variation in BMI (F-statistic = 316; *n* = 32,391). The synthesis allele score explained 0.64% (F-statistic = 230; *n* = 35,873) and the metabolism allele score 1.26% (F statistic = 489; *n* = 38,191) of the variation in 25(OH)D. There was no evidence for variation in the BMI allele score–BMI association by continent (*p*_meta-regression_ = 0.15) or BMI (*p*_meta-regression_ = 0.83). However, the BMI allele score–BMI association was slightly weaker in studies with older compared to younger participants (−0.03% [−0.05% to −0.002%], *p*_meta-regression_ = 0.03). The vitamin D allele score–25(OH)D association did not vary by age, BMI, continent, or assay (*p*_meta-regression_≥0.09 for all comparisons).

#### Evaluation of the genetic outcome associations

Of the 12 individual BMI SNPs, the SNP for *FTO* was the only one that showed evidence of a univariate association with 25(OH)D (*p* = 0.050) (Figure S6). None of the four 25(OH)D SNPs were individually associated with BMI (*p*≥0.10) (Figure S7). The lack of association of the four vitamin D SNPs with BMI was further confirmed using the summary data from the GIANT consortium (*p*>0.30 for all the SNPs) (Table 3).

The association between BMI allele score and 25(OH)D did not vary by study level factors, including age (*p_meta-regression_* = 0.40), BMI (*p_meta-regression_* = 0.18), continent of study (*p_meta-regression_* = 0.78), or vitamin D assay (*p_meta-regression_* = 0.23). Similarly, there was no evidence for variation in the vitamin D allele score–BMI association by age (*p_meta-regression_*≥0.25 for both scores), or continent (*p_meta-regression_*≥0.50 for both scores). There was also no strong evidence for variation in the vitamin D allele score–BMI association by average BMI of the study (≤25 kg/m^2^ versus ≥25 kg/m^2^), although for the synthesis score the meta-regression coefficient was of borderline significance (*p_meta-regression_* = 0.053, Figure S8; *p_meta-regression_* = 0.78 for metabolism score).

#### Power comparison

Illustrative power calculations are presented in Figure S9. In theory, we had greater power to detect an association between 25(OH)D and BMI using the metabolism score as an instrument, compared with an equal sized association between BMI and 25(OH)D using the BMI risk score. However, if the size of the association between 25(OH)D and BMI was only half that seen between BMI and 25(OH)D, our study would not have been adequately powered even with the inclusion of the GIANT results.

## Discussion

Obesity, and perhaps vitamin D deficiency, are among the most important modifiable risk factors for a number of chronic diseases. Obesity and vitamin D status are known to be associated but the direction of the association and whether it is causal has been uncertain. We have presented genetic evidence that higher BMI leads to lower vitamin D status. Conversely, our analyses provided no evidence for a causal role of vitamin D in the development of obesity, although our study was not powered to detect very small effects. These results suggest that although increases in vitamin D status are not likely to help with weight regulation, increased risk of vitamin D deficiency could contribute to the adverse health effects associated with obesity.

The association between obesity and vitamin D status was remarkably consistent across the different populations included in our meta-analyses, being apparent both in men and in women, and in the young and older cohorts alike. Interestingly, the association between obesity and 25(OH)D concentrations appeared stronger for populations in North America compared to Europe, possibly reflecting differences in the distribution of BMI across the continents. Recent intervention studies have shown that obese individuals need higher vitamin D dosages than lean individuals to achieve the same 25(OH)D concentrations [40],[41]. Given that North America has one of the highest rates of obesity in the world [42], our study highlights the importance of considering obesity as a risk factor for vitamin D deficiency with implications on the dosage requirements and possible targeting of relevant health promotion strategies.

The lack of any suggestion for an association between the vitamin D SNPs and BMI in the GIANT consortium (*n* = 123,864) alongside our own large meta-analyses provides a strong case against linear increases in 25(OH)D having a substantive influence on BMI. This conclusion is in accordance with a recent study on Chinese women (*n* = 7,000), which also failed to observe evidence for an association with BMI for genetic variants in the vitamin D pathway [43]. Although a recent RCT (*n* = 77) suggested greater loss in fat mass for women receiving vitamin D [15], previous trials have failed to show any evidence for an effect despite larger treatment groups (*n* = 200–445), use of higher vitamin D dosages, and equal duration of treatment (12 mo) [13],[14]. Dilution related to the greater volume of distribution has been recently proposed as the most likely explanation for the lower 25(OH)D concentrations in obese individuals [44]. In that study, no evidence was found for reduced bioavailability through increased sequestration of vitamin D in the adipose tissue, which had previously been suggested to contribute to the low 25(OH)D concentrations in obesity [18]. In contrast, intact parathyroid hormone (iPTH) levels [45], which stimulate the 1-α-hydroxylase (CYP27B1) enzyme that converts 25(OH)D to 1,25-dihydroxyvitamin D (the active hormonal form), have been found to be elevated in obesity [46], which could to some extent also contribute to the lower 25(OH)D concentrations in obese individuals. It is also possible that differences in lifestyle could contribute to lower 25(OH)D concentrations in obese compared to normal weight individuals, although the association between obesity and low 25(OH)D concentrations has been found to only modestly attenuate after adjustment for vitamin D-related lifestyle and dietary factors [9].

The main strengths of this study are the large sample size and the individual level population-based data from North America and Europe. We used a bi-directional MR approach to investigate the causal directions between obesity and vitamin D deficiency, observing evidence for reductions in 25(OH)D by BMI but not vice versa. However, based on the biological pathways proposed, a possible effect of 25(OH)D on BMI could be expected to be weaker than the effect of BMI on 25(OH)D. Despite including data from the large GIANT consortium to narrow the range of effects compatible with the data, we are unable to exclude very small effects. Furthermore, while the MR approach enables the approximation of life-long differences in average concentrations, with genetic markers it is not possible to examine the influences arising from the extremes of non-linear distributions [20]. Consequently, we cannot discount a possible effect of severe vitamin D deficiency on BMI due to evidence of non-linearity seen in some studies [47]. In contrast, associations between BMI and 25(OH)D within levels in the obesity range were consistently linear in studies included in our analyses (unpublished data), hence the observed association between higher BMI and lower 25(OH)D is likely to be informative in the context of obesity.

One of the methodological challenges of the MR approach relates to the large sample size requirement, arising from the availability of relatively weak instruments for most exposures [22],[31]. This aspect of the MR approach is also reflected in our study, notably in the relatively small amount of variation explained by all the instruments used. We used the IV ratio method on meta-analyzed coefficients since all studies were not able to share individual level participant data. This method assumes linear relationships and may have less power to detect an effect than other IV methods [48]. However, as shown by the clear outcome of these analyses, we were able to overcome these issues by combining several cohorts with comparable information, allowing us to achieve the large numbers required (maximum *n* = 42,024) [31]. To confirm the lack of association between vitamin D-related genetic variations and BMI, we were able to expand the analyses by using data from the large GIANT meta-analyses (*n = *123,864) [23]. However, this cannot be considered an independent replication, as eight of the studies that were part of the D-CarDia Collaboration were also included in GIANT. The F-statistic is used to measure the strength of an instrument, and an instrument that has a value greater than 10 is considered strong enough to use in IV analyses [49]. In our analyses, the F-statistic was greater than 200 for all instruments used due to our large sample size.

Combining large population-based studies from North America and Europe could lead to confounding by population stratification; however, we adjusted for geographical variation/principal components in all analyses, which appeared adequate, as there was no evidence for heterogeneity by continent for the allele score meta-analyses. An important benefit of the MR approach is that it helps to overcome problems of confounding and reverse causality, which limit the ability to draw causal inferences in non-genetic observational studies [19],[20]. However, it could be argued that as the biological function for some of the BMI SNPs is yet to be established, there could be alternative biological pathways explaining their association with BMI. Using multiple SNPs to index BMI, we were able to minimise the risk of pleiotropic effects, as the effects of alternative pathways reflected by individual SNPs would be expected to be strongly diluted when combined in a multi marker score [21],[22].

In conclusion, we demonstrated that the association between BMI and lower 25(OH)D concentrations in Caucasian populations from North America and Europe can be seen across different age groups and in both men and women. We also show that higher BMI leads to lower vitamin D status, providing evidence for the role of obesity as a causal risk factor for the development of vitamin D deficiency. Together with the suggested increases in vitamin D requirements in obese individuals [45],[50], our study highlights the importance of monitoring and treating vitamin D deficiency as a means of alleviating the adverse influences of excess adiposity on health. Our findings suggest that population level interventions to reduce obesity would be expected to lead to a reduction in the prevalence of vitamin D deficiency.

## Supporting Information

Figure S1 **Vitamin D pathway showing the “synthesis” and “metabolism” indicators.**
(TIF)

Figure S2 **Meta-analysis of BMI allele score association with BMI in collaborating studies (** ***n*** **= 32,391).**
(TIF)

Figure S3 **Meta-analysis of synthesis (A) (** ***n*** **= 35,873) and metabolism (B) (** ***n*** **= 38,191) allele score associations with 25(OH)D in collaborating studies.**
(TIF)

Figure S4 **Association of the 12 BMI-related SNPs with BMI.**
(TIF)

Figure S5 **Association of the four vitamin D SNPs with 25(OH)D.**
(TIF)

Figure S6 **Association of the 12 BMI-related SNPs with 25(OH)D.**
(TIF)

Figure S7 **Association of the four vitamin D SNPs with BMI.**
(TIF)

Figure S8 **Meta-analysis of synthesis allele score association with BMI stratified by mean BMI of collaborating studies (** ***n*** **= 36,553).**
(TIF)

Figure S9 **Power to detect an association between BMI and 25(OH)D using genetic proxies in instrumental variable regression.** The solid lines represent the power to detect the same effect size for a BMI association with 25(OH)D using BMI risk score (black line), 25(OH)D association with BMI using metabolism score (mid grey line), and 25(OH)D association with BMI using synthesis score (dark grey line). The dash lines represent the power to detect an effect size half that of the same coloured solid lines for the 25(OH)D association with BMI.
(TIF)

Table S1 **Minor allele frequency (MAF) for the BMI and vitamin D-related SNPs.**
(DOCX)

Text S1 **STROBE statement—checklist of items.**
(DOC)

Text S2 **Study details of the 21 participating cohorts and the funding information.**
(DOCX)

## Abbreviations

25(OH)D: 25-hydroxyvitamin D
BMI: body mass index
GIANT: Genetic Investigation of Anthropometric Traits
IV: instrumental variable
MR: Mendelian randomization

## References

[1] Baskin ML, Ard J, Franklin F, Allison DB (2005) Prevalence of obesity in the United States. Obes Rev 6: 5–7. 15655032 10.1111/j.1467-789X.2005.00165.x

[2] Ogden CL, Carroll MD, Curtin LR, Lamb MM, Flegal KM (2010) Prevalence of high body mass index in US children and adolescents, 2007–2008. JAMA 303: 242–249. 20071470 10.1001/jama.2009.2012

[3] Berghofer A, Pischon T, Reinhold T, Apovian CM, Sharma AM, et al (2008) Obesity prevalence from a European perspective: a systematic review. BMC Public Health 8: 200. 18533989 10.1186/1471-2458-8-200 PMC2441615

[4] Zheng W, McLerran DF, Rolland B, Zhang X, Inoue M, et al (2011) Association between body-mass index and risk of death in more than 1 million Asians. N Engl J Med 364: 719–729. 21345101 10.1056/NEJMoa1010679 PMC4008249

[5] Flegal KM, Carroll MD, Kit BK, Ogden CL (2012) Prevalence of obesity and trends in the distribution of body mass index among US adults, 1999–2010. JAMA 307: 491–497. 22253363 10.1001/jama.2012.39

[6] Vimaleswaran KS, Loos RJ (2010) Progress in the genetics of common obesity and type 2 diabetes. Expert Rev Mol Med 12: e7. 20184785 10.1017/S1462399410001389

[7] Ginde AA, Liu MC, Camargo CA Jr (2009) Demographic differences and trends of vitamin D insufficiency in the US population, 1988–2004. Arch Intern Med 169: 626–632. 19307527 10.1001/archinternmed.2008.604 PMC3447083

[8] Lanham-New SA, Buttriss JL, Miles LM, Ashwell M, Berry JL, et al (2011) Proceedings of the Rank Forum on vitamin D. Br J Nutr 105: 144–156. 21134331 10.1017/S0007114510002576 PMC3408594

[9] Hyppönen E, Power C (2007) Hypovitaminosis D in British adults at age 45 y: nationwide cohort study of dietary and lifestyle predictors. Am J Clin Nutr 85: 860–868. 17344510 10.1093/ajcn/85.3.860

[10] Earthman CP, Beckman LM, Masodkar K, Sibley SD (2012) The link between obesity and low circulating 25-hydroxyvitamin D concentrations: considerations and implications. Int J Obes (Lond) 36: 387–396. 21694701 10.1038/ijo.2011.119

[11] Shi H, Norman AW, Okamura WH, Sen A, Zemel MB (2001) 1alpha,25-Dihydroxyvitamin D3 modulates human adipocyte metabolism via nongenomic action. Faseb J 15: 2751–2753. 11606486 10.1096/fj.01-0584fje

[12] Fassina G, Maragno I, Dorigo P, Contessa AR (1969) Effect of vitamin D2 on hormone-stimulated lipolysis in vitro. Eur J Pharmacol 5: 286–290. 4306335 10.1016/0014-2999(69)90150-2

[13] Sneve M, Figenschau Y, Jorde R (2008) Supplementation with cholecalciferol does not result in weight reduction in overweight and obese subjects. Eur J Endocrinol 159: 675–684. 19056900 10.1530/EJE-08-0339

[14] Zittermann A, Frisch S, Berthold HK, Gotting C, Kuhn J, et al (2009) Vitamin D supplementation enhances the beneficial effects of weight loss on cardiovascular disease risk markers. Am J Clin Nutr 89: 1321–1327. 19321573 10.3945/ajcn.2008.27004

[15] Salehpour A, Shidfar F, Hosseinpanah F, Vafa M, Razaghi M, et al (2012) Vitamin D3 and the risk of CVD in overweight and obese women: a randomised controlled trial. Br J Nutr 1–8. 10.1017/S0007114512000098 22317756

[16] Soares MJ, Murhadi LL, Kurpad AV, Chan She Ping-Delfos WL, Piers LS (2012) Mechanistic roles for calcium and vitamin D in the regulation of body weight. Obes Rev 13: 592–605. 22385576 10.1111/j.1467-789X.2012.00986.x

[17] Foss YJ (2009) Vitamin D deficiency is the cause of common obesity. Med Hypotheses 72: 314–321. 19054627 10.1016/j.mehy.2008.10.005

[18] Wortsman J, Matsuoka LY, Chen TC, Lu Z, Holick MF (2000) Decreased bioavailability of vitamin D in obesity. Am J Clin Nutr 72: 690–693. 10966885 10.1093/ajcn/72.3.690

[19] Davey Smith G, Ebrahim S (2003) ‘Mendelian randomization’: can genetic epidemiology contribute to understanding environmental determinants of disease? Int J Epidemiol 32: 1–22. 12689998 10.1093/ije/dyg070

[20] Lawlor DA, Harbord RM, Sterne JA, Timpson N, Davey Smith G (2008) Mendelian randomization: using genes as instruments for making causal inferences in epidemiology. Stat Med 27: 1133–1163. 17886233 10.1002/sim.3034

[21] Davey Smith G (2011) Random allocation in observational data: how small but robust effects could facilitate hypothesis-free causal inference. Epidemiology 22: 460–463; discussion 467–468. 21642771 10.1097/EDE.0b013e31821d0426

[22] Palmer TM, Lawlor DA, Harbord RM, Sheehan NA, Tobias JH, et al (2012) Using multiple genetic variants as instrumental variables for modifiable risk factors. Stat Methods Med Res 21: 223–242. 21216802 10.1177/0962280210394459 PMC3917707

[23] Speliotes EK, Willer CJ, Berndt SI, Monda KL, Thorleifsson G, et al (2010) Association analyses of 249,796 individuals reveal 18 new loci associated with body mass index. Nat Genet 42: 937–948. 20935630 10.1038/ng.686 PMC3014648

[24] Li S, Zhao JH, Luan J, Luben RN, Rodwell SA, et al (2010) Cumulative effects and predictive value of common obesity-susceptibility variants identified by genome-wide association studies. Am J Clin Nutr 91: 184–190. 19812171 10.3945/ajcn.2009.28403

[25] Loos RJ, Lindgren CM, Li S, Wheeler E, Zhao JH, et al (2008) Common variants near MC4R are associated with fat mass, weight and risk of obesity. Nat Genet 40: 768–775. 18454148 10.1038/ng.140 PMC2669167

[26] Thorleifsson G, Walters GB, Gudbjartsson DF, Steinthorsdottir V, Sulem P, et al (2009) Genome-wide association yields new sequence variants at seven loci that associate with measures of obesity. Nat Genet 41: 18–24. 19079260 10.1038/ng.274

[27] Wang TJ, Zhang F, Richards JB, Kestenbaum B, van Meurs JB, et al (2010) Common genetic determinants of vitamin D insufficiency: a genome-wide association study. Lancet 376: 180–188. 20541252 10.1016/S0140-6736(10)60588-0 PMC3086761

[28] Zheng J, Li Y, Abecasis GR, Scheet P (2011) A comparison of approaches to account for uncertainty in analysis of imputed genotypes. Genet Epidemiol 35: 102–110. 21254217 10.1002/gepi.20552 PMC3143715

[29] Cole TJ (2000) Sympercents: symmetric percentage differences on the 100 log(e) scale simplify the presentation of log transformed data. Stat Med 19: 3109–3125. 11113946 10.1002/1097-0258(20001130)19:22<3109::aid-sim558>3.0.co;2-f

[30] Lin X, Song K, Lim N, Yuan X, Johnson T, et al (2009) Risk prediction of prevalent diabetes in a Swiss population using a weighted genetic score–the CoLaus Study. Diabetologia 52: 600–608. 19139842 10.1007/s00125-008-1254-y

[31] Pierce BL, Ahsan H, Vanderweele TJ (2011) Power and instrument strength requirements for Mendelian randomization studies using multiple genetic variants. Int J Epidemiol 40: 740–752. 20813862 10.1093/ije/dyq151 PMC3147064

[32] Berry DJ, Vimaleswaran KS, Whittaker JC, Hingorani AD, Hypponen E (2012) Evaluation of genetic markers as instruments for mendelian randomization studies on vitamin D. PLoS One 7: e37465 doi:10.1371/journal.pone.0037465. 22629401 10.1371/journal.pone.0037465 PMC3357436

[33] Chun RF, Lauridsen AL, Suon L, Zella LA, Pike JW, et al (2010) Vitamin D-binding protein directs monocyte responses to 25-hydroxy- and 1,25-dihydroxyvitamin D. J Clin Endocrinol Metab 95: 3368–3376. 20427486 10.1210/jc.2010-0195 PMC2928899

[34] Rice JA (1995) Expected values. Mathematical statistics and data analysis. 2nd edition. Pacific Grove (California): Duxbury Press.

[35] Ehret GB, Munroe PB, Rice KM, Bochud M, Johnson AD, et al (2011) Genetic variants in novel pathways influence blood pressure and cardiovascular disease risk. Nature 478: 103–109. 21909115 10.1038/nature10405 PMC3340926

[36] Thomas DC, Lawlor DA, Thompson JR (2007) Re: Estimation of bias in nongenetic observational studies using “Mendelian triangulation” by Bautista et al. Ann Epidemiol 17: 511–513. 17466535 10.1016/j.annepidem.2006.12.005

[37] White IR (2009) Multivariate random-effects meta-analysis. The Stata Journal 9: 40–56.

[38] Borenstein M (2009) Introduction to meta-analysis. Chichester: John Wiley & Sons. xxviii.

[39] StataCorp (2011). Stata Statistical Software: Release 12: College Station (Texas): StataCorp LP.

[40] Jorde R, Sneve M, Emaus N, Figenschau Y, Grimnes G (2010) Cross-sectional and longitudinal relation between serum 25-hydroxyvitamin D and body mass index: the Tromso study. Eur J Nutr 49: 401–407. 20204652 10.1007/s00394-010-0098-7

[41] Lee P, Greenfield JR, Seibel MJ, Eisman JA (2009) Center JR (2009) Adequacy of vitamin D replacement in severe deficiency is dependent on body mass index. Am J Med 122: 1056–1060. 19854337 10.1016/j.amjmed.2009.06.008

[42] Bassett DR Jr, Pucher J, Buehler R, Thompson DL, Crouter SE (2008) Walking, cycling, and obesity rates in Europe, North America, and Australia. J Phys Act Health 5: 795–814. 19164816 10.1123/jpah.5.6.795

[43] Dorjgochoo T, Shi J, Gao YT, Long J, Delahanty R, et al (2012) Genetic variants in vitamin D metabolism-related genes and body mass index: analysis of genome-wide scan data of approximately 7000 Chinese women. Int J Obes (Lond) 36: 1252–1255. 22158264 10.1038/ijo.2011.246 PMC3779367

[44] Drincic AT, Armas LA, Van Diest EE, Heaney RP (2012) Volumetric dilution, rather than sequestration best explains the low vitamin D status of obesity. Obesity (Silver Spring) 20: 1444–1448. 22262154 10.1038/oby.2011.404

[45] Bell NH, Epstein S, Greene A, Shary J, Oexmann MJ, et al (1985) Evidence for alteration of the vitamin D-endocrine system in obese subjects. J Clin Invest 76: 370–373. 2991340 10.1172/JCI111971 PMC423785

[46] Holick MF (2007) Vitamin D deficiency. N Engl J Med 357: 266–281. 17634462 10.1056/NEJMra070553

[47] Hyppönen E, Berry D, Cortina-Borja M, Power C (2010) 25-Hydroxyvitamin D and pre-clinical alterations in inflammatory and hemostatic markers: a cross sectional analysis in the 1958 British Birth Cohort. PLoS One 5: e10801 doi:10.1371/journal.pone.0010801. 20520739 10.1371/journal.pone.0010801 PMC2875406

[48] Burgess S, Thompson SG, Andrews G, Samani NJ, Hall A, et al (2010) Bayesian methods for meta-analysis of causal relationships estimated using genetic instrumental variables. Stat Med 29: 1298–1311. 20209660 10.1002/sim.3843 PMC3648673

[49] Staiger D, Stock JH (1997) Instrumental variables regression with weak instruments. Econometrica 65: 557–586.

[50] Huh SY, Gordon CM (2008) Vitamin D deficiency in children and adolescents: epidemiology, impact and treatment. Rev Endocr Metab Disord 9: 161–170. 18175220 10.1007/s11154-007-9072-y

