# Supplementary figures and images for: Causal Relationship between Obesity and Vitamin D Status: Bi-Directional Mendelian Randomization Analysis of Multiple Cohorts
Source: PLoS Med. 2013 Feb 5;10(2):e1001383. doi: 10.1371/journal.pmed.1001383 (PMC3564800; doi:10.1371/journal.pmed.1001383)

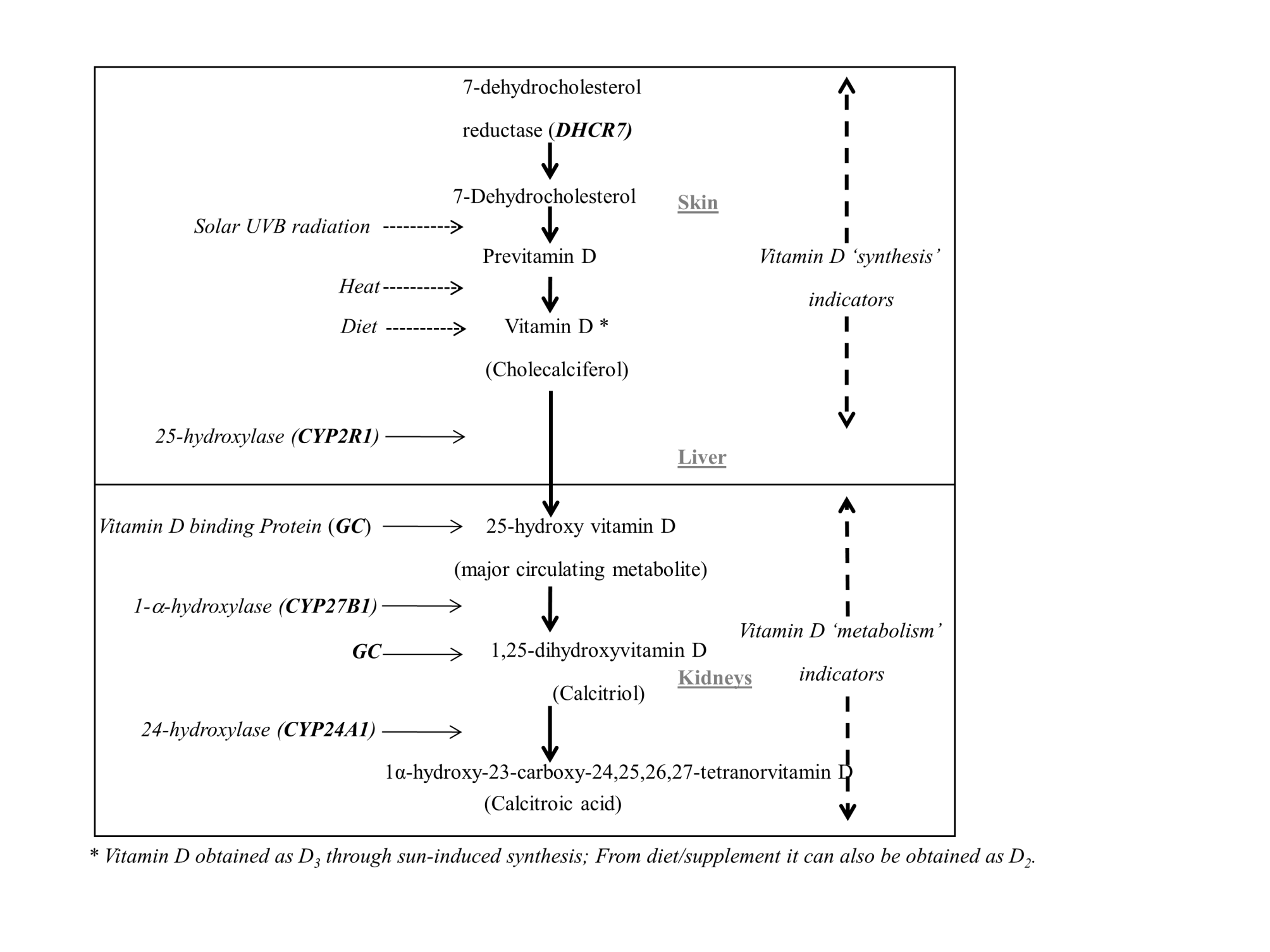

Supplement: Figure S1 — Vitamin D pathway showing the “synthesis” and “metabolism” indicators. (TIF) [file pmed.1001383.s001.tif]

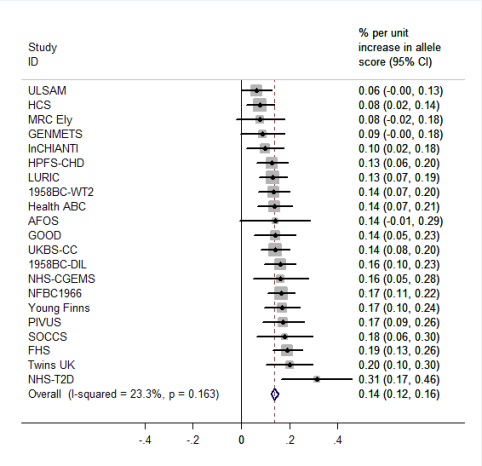

Supplement: Figure S2 — Meta-analysis of BMI allele score association with BMI in collaborating studies ( n = 32,391). (TIF) [file pmed.1001383.s002.tif]

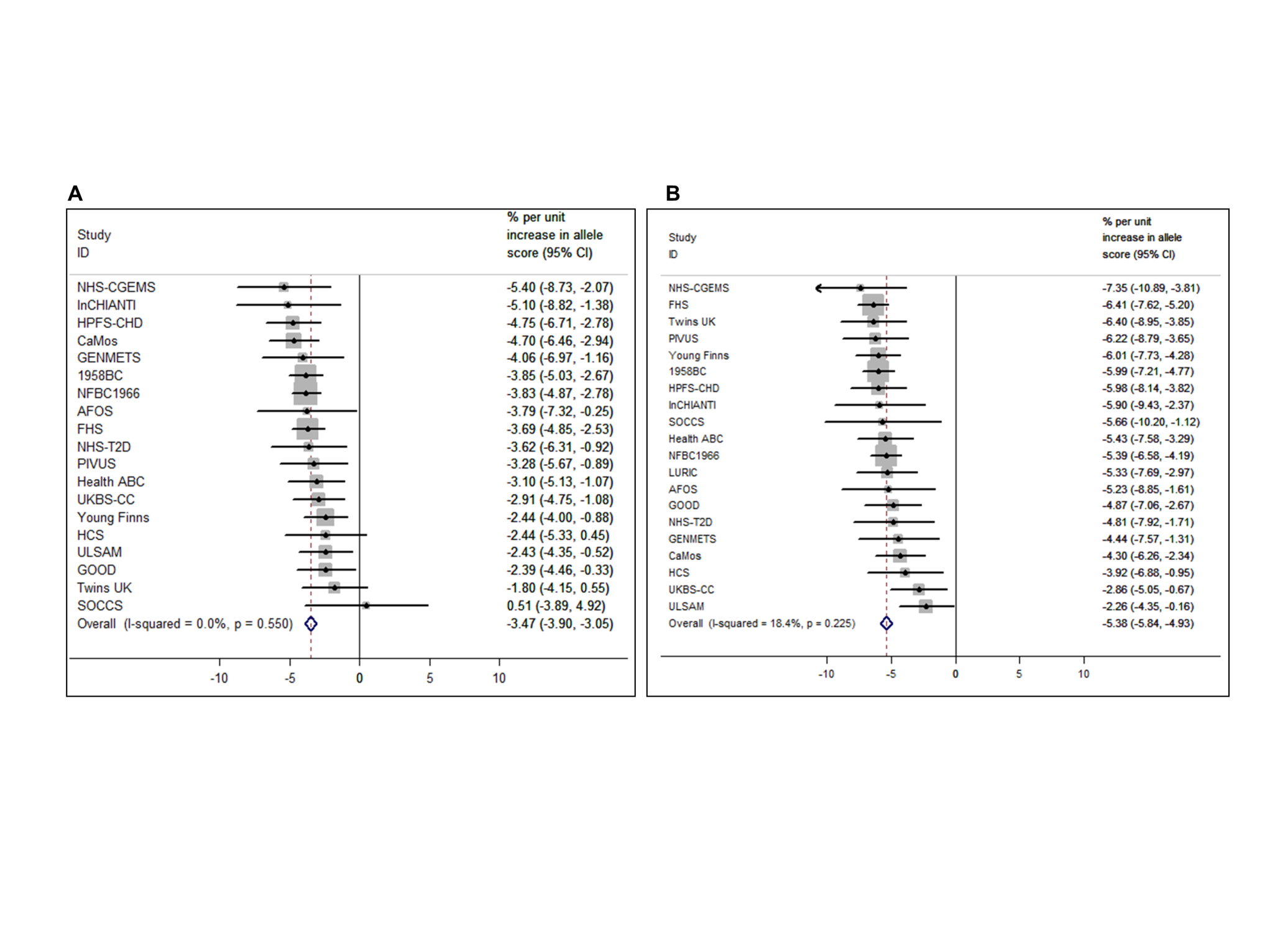

Supplement: Figure S3 — Meta-analysis of synthesis (A) ( n = 35,873) and metabolism (B) ( n = 38,191) allele score associations with 25(OH)D in collaborating studies. (TIF) [file pmed.1001383.s003.tif]

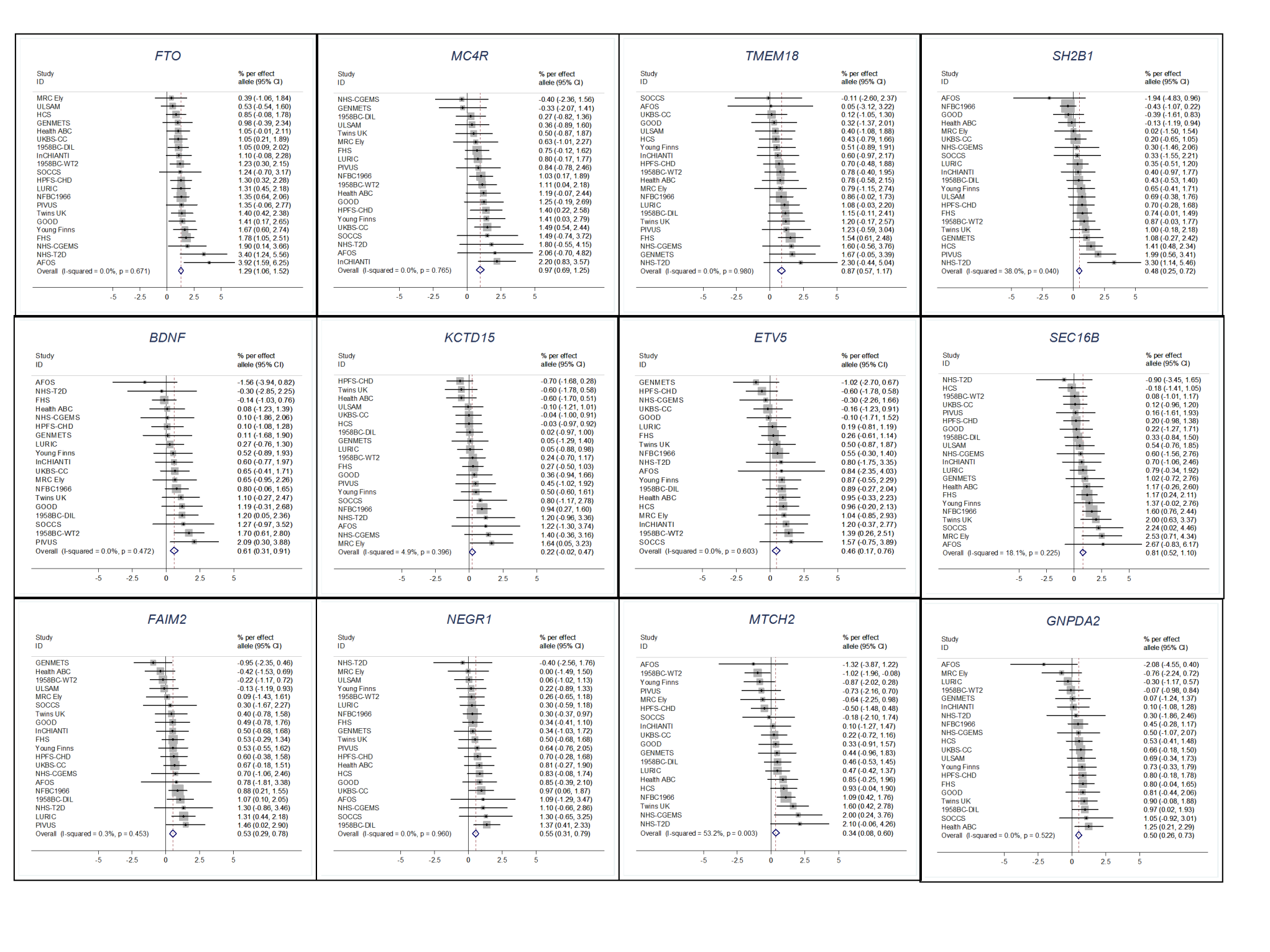

Supplement: Figure S4 — Association of the 12 BMI-related SNPs with BMI. (TIF) [file pmed.1001383.s004.tif]

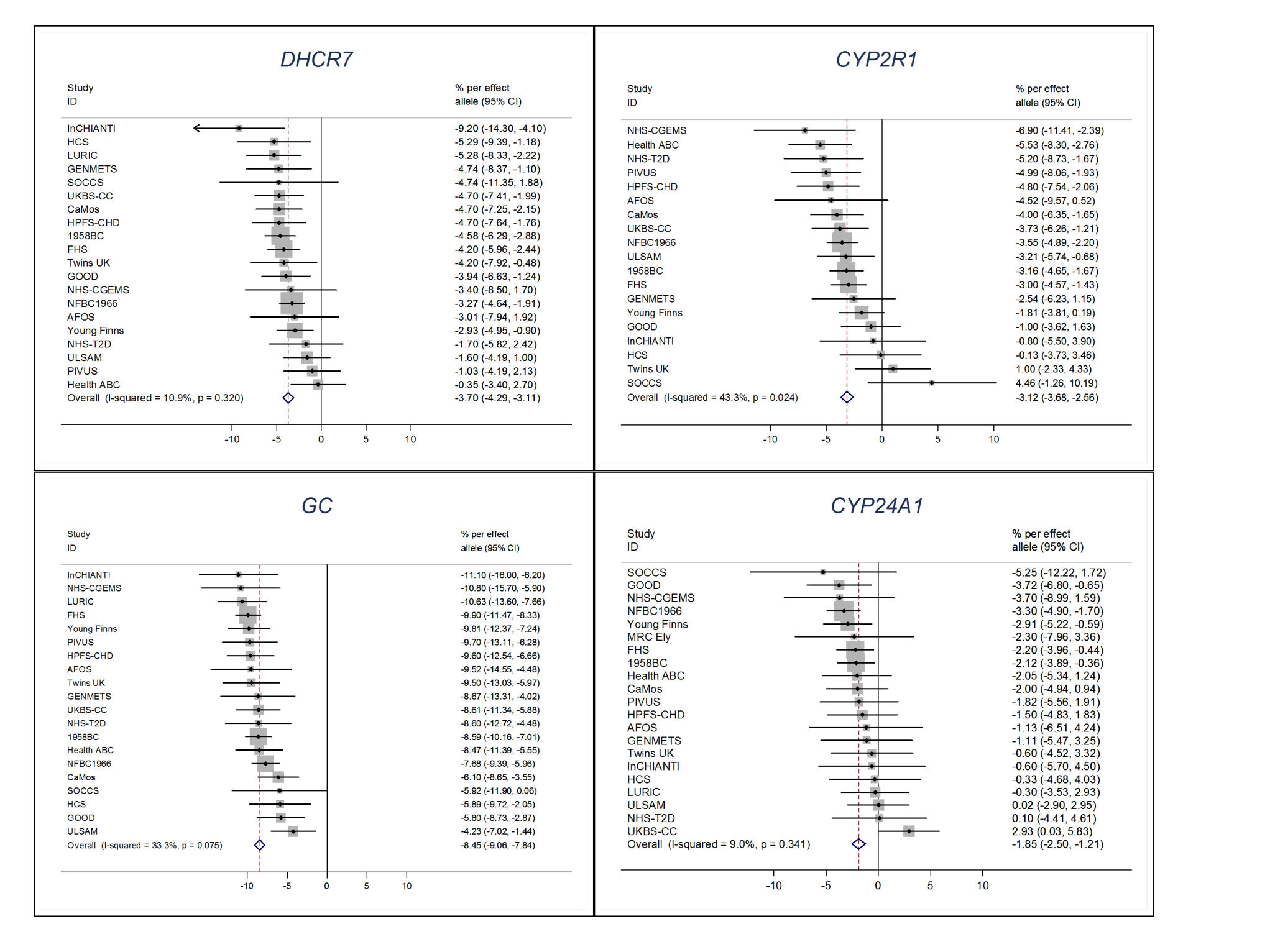

Supplement: Figure S5 — Association of the four vitamin D SNPs with 25(OH)D. (TIF) [file pmed.1001383.s005.tif]

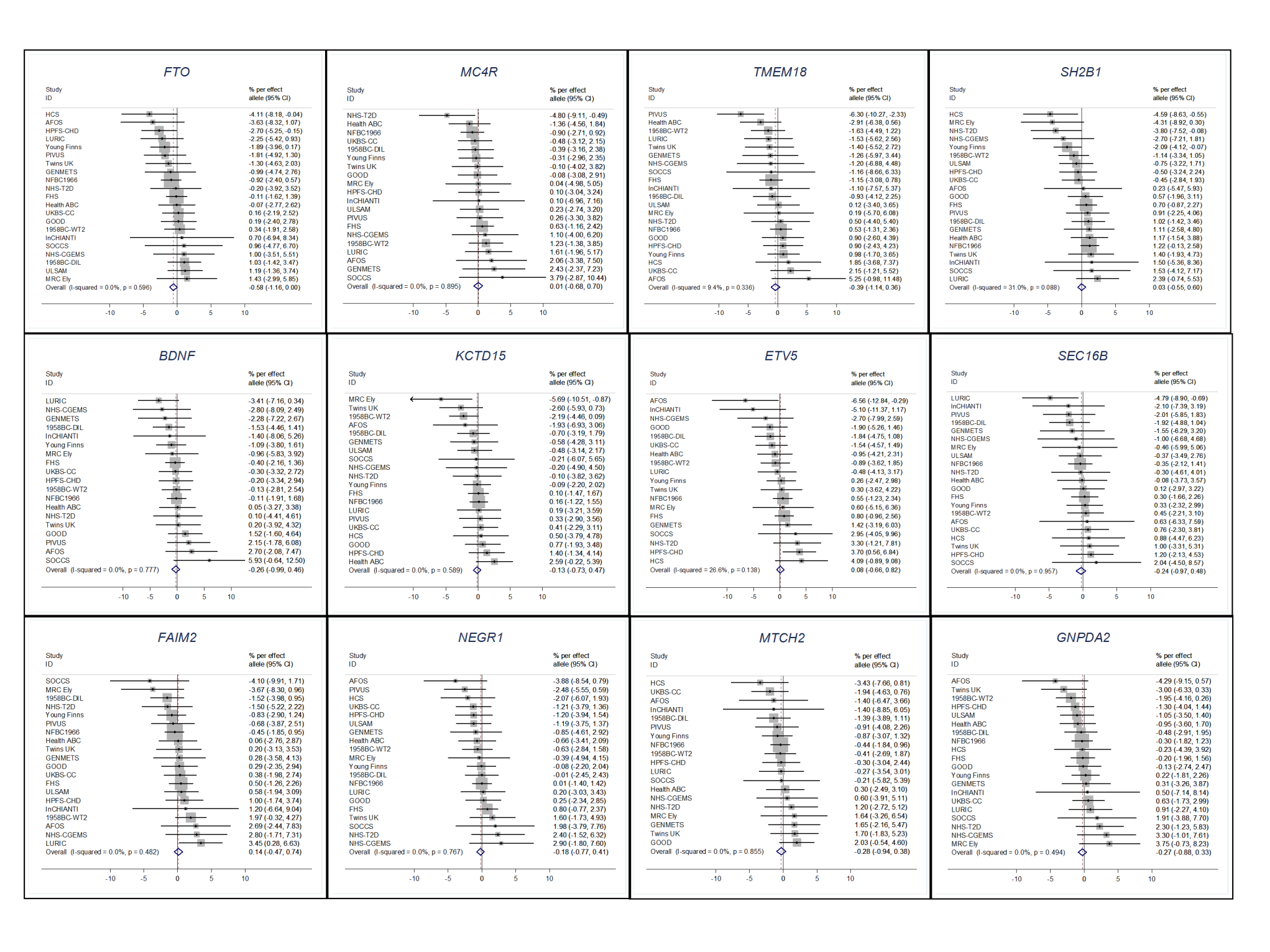

Supplement: Figure S6 — Association of the 12 BMI-related SNPs with 25(OH)D. (TIF) [file pmed.1001383.s006.tif]

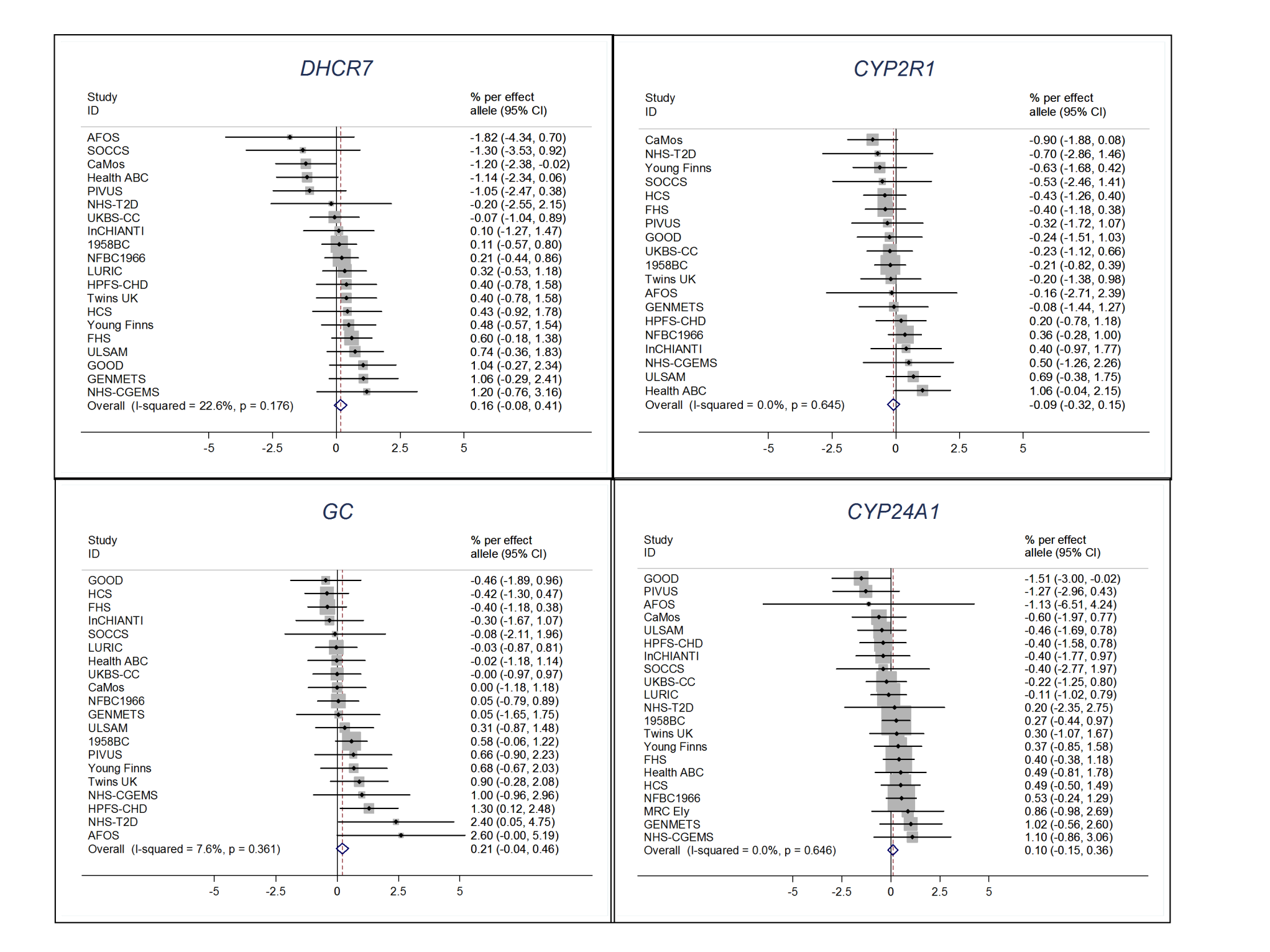

Supplement: Figure S7 — Association of the four vitamin D SNPs with BMI. (TIF) [file pmed.1001383.s007.tif]

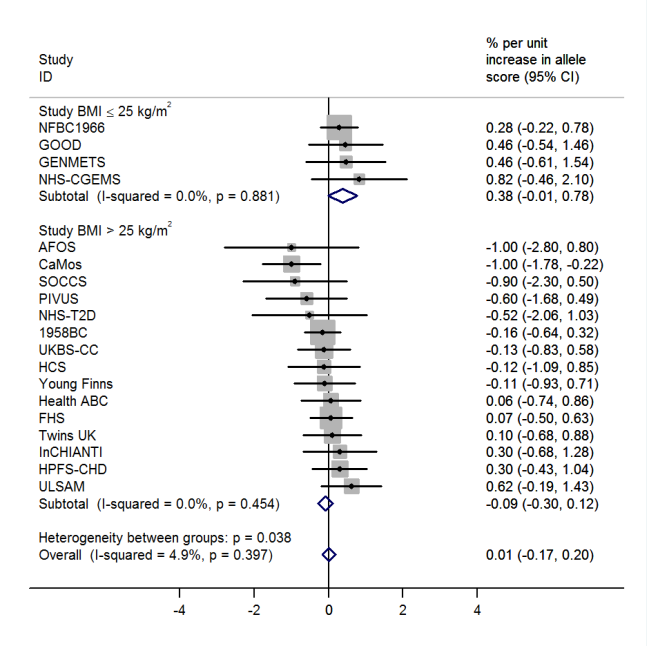

Supplement: Figure S8 — Meta-analysis of synthesis allele score association with BMI stratified by mean BMI of collaborating studies ( n = 36,553). (TIF) [file pmed.1001383.s008.tif]

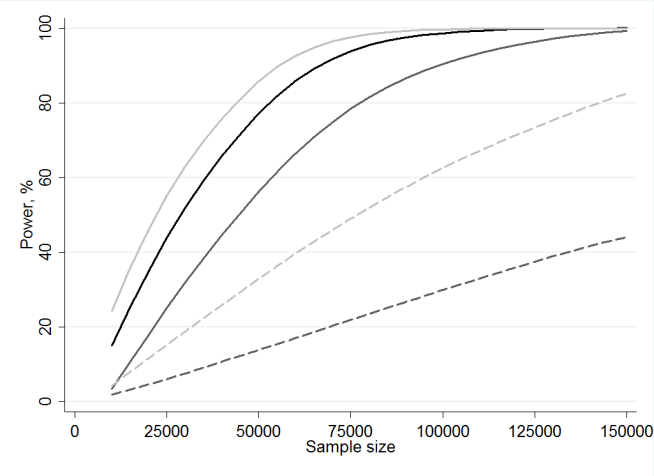

Supplement: Figure S9 — Power to detect an association between BMI and 25(OH)D using genetic proxies in instrumental variable regression. The solid lines represent the power to detect the same effect size for a BMI association with 25(OH)D using BMI risk score (black line), 25(OH)D association with BMI using metabolism score (mid grey line), and 25(OH)D association with BMI using synthesis score (dark grey line). The dash lines represent the power to detect an effect size half that of the same coloured solid lines for the 25(OH)D association with BMI. (TIF) [file pmed.1001383.s009.tif]
