# Supplementary material for: Causal Relationship between Obesity and Vitamin D Status: Bi-Directional Mendelian Randomization Analysis of Multiple Cohorts
Source: PLoS Med. 2013 Feb 5;10(2):e1001383. doi: 10.1371/journal.pmed.1001383 (PMC3564800; doi:10.1371/journal.pmed.1001383)
Supplement: Table S1 — Minor allele frequency (MAF) for the BMI and vitamin D-related SNPs. (DOCX) [file pmed.1001383.s010.docx]

**Table S1**: **Minor allele frequency (MAF) for the body mass index and vitamin D related single nucleotide polymorphisms (SNPs)**

| **Gene name** | **SNP rs no** | **Effect allele** | **1958BC** | **AFOS** | **CaMos*** | | **NHS-CGEMS** | **HPFS-CHD** | **NHS-T2D** | **FHS** | **Health ABC** | **GENMETS** | **HCS** | **InCHIANTI** |
| --- | --- | --- | --- | --- | --- | --- | --- | --- | --- | --- | --- | --- | --- | --- |
|  |  |  | **MAF (%)** | **MAF (%)** | **MAF (%)** | | **MAF (%)** | **MAF (%)** | **MAF (%)** | **MAF (%)** | **MAF (%)** | **MAF (%)** | **MAF (%)** | **MAF (%)** |
| **BMI-related SNPs** | | | | | | | | | | | | | | |
| *FTO* | rs9939609 | A | 39.1 | 46.5 | | - | 39.7 | 42.4 | 40.6 | 40.4 | 42.3 | 37.6 | 43.6 | 43.9 |
| *MC4R* | rs17782313 | C | 23.6 | 32.9 | | - | 24 | 23 | 24.7 | 21.8 | 23 | 17.3 | - | 28.2 |
| *TMEM18* | rs2867125 | C | 17.4 | 16.8 | | - | 18 | 18.2 | 16.3 | 17.8 | 17.9 | 17.1 | 16.7 | 22.2 |
| *SH2B1* | rs7498665 | G | 41.0 | 22 | | - | 41.1 | 38 | 39 | 35.4 | 37.3 | 40.0 | 38.6 | 27.9 |
| *BDNF* | rs4074134 | C | 20.4 | 38.6 | | - | 20.3 | 22.2 | 21.6 | 20.8 | 20.8 | 15.6 | - | 25.3 |
| *KCTD15* | rs29941 | G | 32.7 | 38.8 | | - | 32.2 | 33.3 | 34.1 | 32.4 | 32.5 | 38.2 | 35.1 | - |
| *ETV5* | rs7647305 | C | 20.9 | 16.1 | | - | 21.4 | 22.4 | 21.4 | 21.9 | 21 | 18.5 | 20.0 | 20.2 |
| *SEC16B* | rs10913469 | C | 21.1 | 14.1 | | - | 18 | 20.1 | 22.4 | 18.5 | 16.5 | 17.5 | 19.9 | 15.2 |
| *FAIM2* | rs7138803 | A | 36.5 | 36.2 | | - | 36.5 | 39.1 | 40.8 | 38.4 | 37.8 | 37.5 | - | 36.7 |
| *NEGR1* | rs3101336 | C | 40.0 | 43.9 | | - | 36.2 | 36.5 | 38.3 | 36.4 | 35.8 | 35.3 | 40.4 | - |
| *MTCH2* | rs10838738 | G | 34.5 | 33.5 | | - | 37.5 | 35.3 | 35.4 | - | 35.8 | 33.7 | 33.2 | 33.6 |
| *GNPDA2* | rs10938397 | G | 43.0 | 42.1 | | - | 43.4 | 43.4 | 44 | 41.2 | 44 | 46.4 | 43.4 | 38.8 |
| **Vitamin D-related SNPs** | | | | | | | | | | | | | | |
| *DHCR7* | rs12785878 | G | 22.0 | 30.8 | | 27.2 | 25.9 | 27.3 | 26 | 25.8 | 27 | 37.7 | 25.6 | 24.8 |
| *CYP2R1* | rs10741657 | G | 39.8 | 33.9 | | 37.8 | 40.7 | 38.7 | 40.9 | 40.3 | 37.4 | 39.5 | 38.5 | 31.1 |
| *GC* | rs2282679 | G | 29.7 | 38.3 | | 28.4 | 28.2 | 27.6 | 28.2 | 28.1 | 28.9 | 18.8 | 29.5 | 25.2 |
| *CYP24A1* | rs6013897 | A | 19.6 | 26.2 | | 19.2 | 22.4 | 20.8 | 21.2 | 21.1 | 21.3 | 22.7 | 20.3 | 23.8 |

**Table S1**: **Minor allele frequency (MAF) for the body mass index and vitamin D related single nucleotide polymorphisms (SNPs) (Cont).**

| **Gene name** | **SNP rs no** | **Effect allele** | **LURIC*** | **MRC Ely*** | **NFBC 1966** | **PIVUS** | **SOCCS** | **GOOD** | **Twins UK** | **UKBS-CC** | **ULSAM** | **Young Finns** |
| --- | --- | --- | --- | --- | --- | --- | --- | --- | --- | --- | --- | --- |
|  |  |  | **MAF (%)** | **MAF (%)** | **MAF (%)** | **MAF (%)** | **MAF (%)** | **MAF (%)** | **MAF (%)** | **MAF (%)** | **MAF (%)** | **MAF (%)** |
| **BMI-related SNPs** | | | | | | | | | | | | |
| *FTO* | rs9939609 | A | 41.9 | 39.7 | 39.0 | 40.4 | 37.2 | 40.7 | 39.7 | 40.6 | 38.9 | 40.2 |
| *MC4R* | rs17782313 | C | 25.9 | 27 | 18.1 | 24.2 | 23.4 | 24.6 | 22.2 | 23.8 | 24.7 | 17.4 |
| *TMEM18* | rs2867125 | C | 18.4 | 17.2 | 15.6 | 17.7 | 18.1 | 16.7 | 17.5 | 16.7 | 15.5 | 16.3 |
| *SH2B1* | rs7498665 | G | 40.7 | 40.3 | 42.5 | 41.6 | 40.7 | 42.5 | 39.8 | 38.4 | 43.4 | 41.4 |
| *BDNF* | rs4074134 | C | 22.7 | 26 | 17.2 | 17.8 | 21.3 | 21.3 | 20.5 | 21.6 | - | 16.5 |
| *KCTD15* | rs29941 | G | 30.6 | 32.5 | 39.6 | 33.3 | 34.7 | 32.4 | 33.5 | 32.1 | 32.1 | 38.8 |
| *ETV5* | rs7647305 | C | 23.6 | 18.7 | 17.3 | - | 22.3 | 17.8 | 21.8 | 22.5 | - | 16.9 |
| *SEC16B* | rs10913469 | C | 17.2 | 19.5 | 17.6 | 22.6 | 21.5 | 21.7 | 18.6 | 20.6 | 20.7 | 16.8 |
| *FAIM2* | rs7138803 | A | 38.3 | 36.5 | 36.0 | 41.7 | 36.5 | 40.4 | 36.9 | 37.3 | 42.0 | 37.8 |
| *NEGR1* | rs3101336 | C | 37.8 | 38.7 | 36.0 | 41.2 | 39.1 | 38.9 | 39.2 | 39.5 | 40.9 | 34 |
| *MTCH2* | rs10838738 | G | 33.2 | 32.6 | 36.4 | 36.6 | 35.8 | 35.6 | 35.3 | 35.1 | - | 34.8 |
| *GNPDA2* | rs10938397 | G | 44.3 | 42.4 | 46.9 | - | 41.4 | 40.7 | 43.6 | 44.2 | 41.9 | 48.4 |
| **Vitamin D-related SNPs** | | | | | | | | | | | | |
| *DHCR7* | rs12785878 | G | 24.0 | 25.0 | 39.2 | 34.8 | 22.2 | 32.9 | 22.8 | 23.0 | 33.3 | 40.2 |
| *CYP2R1* | rs10741657 | G | - | - | 42.8 | 43.2 | 40.5 | 40.2 | 40.6 | 40.4 | 43.3 | 42.9 |
| *GC* | rs2282679 | G | 28.1 | - | 19.5 | 26.3 | 28.7 | 25.0 | 29.8 | 29.2 | 26.3 | 18.9 |
| *CYP24A1* | rs6013897 | A | 21.0 | 20.7 | 22.8 | 21.4 | 20 | 20.7 | 19.9 | 19.4 | 22.0 | 23.8 |

*FTO*, Fat mass and obesity associated; *MC4R*, Melanocortin 4 receptor; *TMEM18*, Transmembrane protein 18; *SH2B1*, SH2B adaptor protein 1; *BDNF*, Brain-derived neurotrophic factor; *KCTD15*, Potassium channel tetramerisation domain containing 15; *ETV5*, ets variant 5; *SEC16B*, SEC16 homolog B; *FAIM2*, Fas apoptotic inhibitory molecule 2; *NEGR1*, Neuronal growth regulator 1; *MTCH2*, Mitochondrial carrier 2; *GNPDA2*, Glucosamine-6-phosphate deaminase

***** MRC Ely study and the CaMos study were not included in the vitamin D allele score and BMI allele score analyses, respectively, as the SNPs required for creating the allele score were not available. For the LURIC study, *CYP2R1* SNP was not available and hence, synthesis allele score was not created.
