# Supplementary material for: Causal Relationship between Obesity and Vitamin D Status: Bi-Directional Mendelian Randomization Analysis of Multiple Cohorts
Source: PLoS Med. 2013 Feb 5;10(2):e1001383. doi: 10.1371/journal.pmed.1001383 (PMC3564800; doi:10.1371/journal.pmed.1001383)
Supplement: Text S2 — Study details of the 21 participating cohorts and the funding information. (DOCX) [file pmed.1001383.s012.docx]

**Supplementary Information**

**Study details**

1. **1958 Birth cohort (1958BC):**

*Data collection*: The British 1958 birth cohort (1958BC) [[1](#_ENREF_1)] includes all births during one week in March in England, Scotland and Wales. Participants in the study have been followed regularly with information collected on a wide-range of factors related to health, lifestyle, growth and development. At age 44-45y (August 2002 to March 2004) cohort members were invited to a biomedical survey, which included measurement of weights and heights from 9,348 participants. 8,432 cohort members consented to genetic studies, of whom 7,414 also had information on serum 25(OH)D concentrations and BMI.

*Measurement of serum 25(OH)D*: Serum 25(OH)D concentrations were measured using automated application of the IDS OCTEIA ELISA on the Dade-Behring BEP2000 analyzer (sensitivity of 5.0nmol/L, linearity ≤155nmol/L, and intra-assay CV 5.5-7.2%), with adjustment according to the mean of the Vitamin D External Quality Assessment Scheme.

*Measurement of BMI*: Weight and standing height (n= 9,348) were measured without shoes and in light clothing by a trained nurse. Body mass index was calculated as weight (kg)/height (m)^2^.

*Genotyping*: 3,000 DNA samples were selected as control subjects in the Welcome Trust Case Control Consortium 2 (WTCCC2). This includes an initial 1,500 sampled in the first round of WTCCC. The samples were genotyped on the Affymetrix 6.0 platform and the genotype calling algorithm was Chiamo [[2](#_ENREF_2)]. Sample exclusions included relatedness, exceeding the heterozygosity thresholds, non-European ancestry, gender discrepancy, outlying allele intensities from a selection of single nucleotide polymorphisms (SNPs) compared to the larger sample and discordance with external genotyping. The SNP exclusions were done for MAF < 1%, statistical information from the genotyping call <0.975, Hardy-Weinberg equilibrium (HWE) p-value <1e-20, SNP missingness >0.02 and gene-chip plate association 1e-5. An additional 2500 participants were genotyped by the Type 1 Diabetes Genetics Consortium on the Illumina 550K Infinium platform and the genotyping calling algorithm was Illuminus [[3](#_ENREF_3)]. Sample exclusions were done on sample call rate > 3%, exceeding the heterozygosity thresholds, gender discrepancy and non-European ancestry. The SNP exclusions were done for MAF < 1%, HWE p-value < 1e-7 and SNP call rate <95%. The vitamin D SNPs were genotyped using the Taqman platform (Applied Biosystems, Warrington, UK).

1. **Amish Family Osteoporosis Study (AFOS):**

*Data collection*: The AFOS recruited Old Order Amish individuals from Lancaster, Pennsylvania, from 1997-2008 [[4](#_ENREF_4)]. The cohort includes 1,520 individuals in multigenerational families, all of whom can be connected in a single pedigree. Participants were at least 20 years old and generally healthy. Exclusion criteria included malignancy, liver and kidney disease (serum creatinine > 1.3 mg/dl), Paget disease of bone, hypocalcaemia, untreated thyroid disease and severe hypertension. The current analysis is restricted to 330 participants who had genome-wide association study (GWAS) data as well as vitamin D and BMI measurements.

*Measurement of serum 25(OH)D*: Circulating concentrations of serum 25(OH)D were measured in aliquots of serum samples stored at -80°C until testing using a radioimmunoassay procedure (DiaSorin, Stillwater, MN). The assay was run in duplicate at The Johns Hopkins Bayview Medical Center General Clinical Research Center (Baltimore, MD) according to the manufacturer's instructions, and the mean of the duplicate values was used. Intra-assay and inter-assay CV were 5.19% and 7.90% respectively.

*Measurement of BMI*: Weight and height measurements were conducted by trained clinic staff while participants wore lightweight clothing without shoes. Weight was measured to the nearest tenth of a kg and height was measured to the nearest millimetre.

*Genotyping*: Genotyping was conducted on either Affymetrix 500K SNP or 6.0 arrays. The GeneChip Genotyping Analysis Software (GTYPE 4.0) was used to generate dynamic modelling algorithm– derived genotypes 10 that were reanalyzed with the BRLMM (Bayesian RLMM) genotype calling algorithm (confidence threshold of 0.33) to improve the proportion of heterozygote calls. As an initial quality-control measure, BRLMM-generated chip files with call rates <90% for both enzymes across all SNPs were excluded. The number of monomorphic and low-frequency SNPs (n=26,816) in the Amish was not appreciably different from that observed in more outbred Caucasians of the HapMap CEU sample assay. One SNP (rs7498665) was genotyped using TaqMan technology (ABI Life Technologies; Carlsbad, CA). Imputation was conducted using MACH.

1. **Canadian Multicentre Osteoporosis Study (CaMos):**

*Data collection*: The CaMos is an ongoing, population-based prospective cohort study [[5](#_ENREF_5)] consisted of all non-institutionalized Canadians living within 50 km of nine urban regional centers. A total of 9,424 randomly selected women (6,539) and men (2,884) aged 25 years and older at baseline (1995-1997), were involved in the study. All available subjects were measured for bone mineral density and vitamin D-related traits at an initial baseline visit and participants in the study were interviewed by a trained interviewer to assess for vitamin D intake, osteoporosis and fracture-related risk factors. A second intensive interview was conducted five and ten years after enrolment to reassess these risk factors. Blood samples for DNA and 25(OH)D analyses were available for the present study from subjects enrolled at seven centres collected at year 10 (n= 2,297).

*Measurement of serum 25(OH)D*: Serum 25(OH)D concentrations were measured by radio-immunoassay (DiaSorin Inc, Stillwater, MN, USA) in CaMos. Detection limits was 4 nmol/L. Inter- and intra-assay variability were 2.9-5.5% and 6.3-12.9%, respectively.

*Measurement of BMI*: Weight and standing height were measured without shoes and in light clothing by a trained nurse. Body mass index was calculated as weight (kg)/height (m)^2^.

*Genotyping*: Genotyping was performed using the Applied Biosystems (ABI) TaqMan protocol and Sequenom protocol. The genotype call rate is > 97%. Concordance rate is 100% for all the SNPs. There were no significant deviations from HWE (p >0.05).

1. **Cancer Genetic Markers of Susceptibility, case control study of breast cancer (NHS-CGEMS)**

*Data collection*: Nurses’ Health Study (NHS) began in 1976 when 121,700 female US registered nurses, 30 to 55 years of age were enrolled [[6](#_ENREF_6)]. The cohort is contacted every 2 years by questionnaire to update diet, medications, anthropometrics and incident physician-diagnosed illnesses with response rates to the follow-up questionnaires are >90% in each cycle. Between 1989 and 1990, 32,826 women between the ages of 43 and 69, have provided blood samples. For the present study, 870 women with GWAS data and serum 25(OH)D were taken from a nested case-control study of breast cancer conducted in NHS [Cancer Genetic Markers of Susceptibility].

*Measurement of serum 25(OH)D*: Serum 25(OH)D concentrations for NHS-CGEMS plasma samples were measured by radioimmunoassay (RIA) [[7](#_ENREF_7)] in three batches, two in Dr. Michael Hollick’s laboratory at Boston University School of Medicine (coefficients for variation (CV) 8.7 – 17.6%) and a third in Dr. Bruce Hollis’ laboratory at the Medical University of South Carolina in Charlestown, SC (CV 8.7%) [[8](#_ENREF_8)].

*Measurement of BMI*: The NHS mailed questionnaire includes items about their medical history including height and weight. These measures taken at the time of blood draw were used to compute BMI defined as weight in kilograms divided by height in meters to the second power. Should these measures not be available at the time of blood draw, the information was taken from the preceding questionnaire (2 years prior).

*Genotyping*: GWAS genotyping used the Illumina 550K platform. Genotypes for markers contained on the Affymetrix 6.0 platform and not on the Illumina 550K platform were imputed using a Markov Chain based Haplotyper (MACH) and HapMap CEU reference panel (Rel 22), with retention of only high quality SNPs with a MACH-r2 of 0.95 or greater in all five data sets. Quality-control assessment of genotypes including sample completion rates, SNP call rates, concordance rates, deviation from Hardy–Weinberg proportions in control DNA and final sample selection for associational analysis are described in detail elsewhere.

1. **Case-control study of coronary heart disease (HPFS-CHD)**

*Data collection*: The Health Professionals Follow-up Study (HPFS) began in 1986 when 51,529 US male dentists, optometrists, osteopaths, podiatrists, pharmacists, and veterinarians, ages 40–75 years, responded to a mailed questionnaire [[9](#_ENREF_9)]. Baseline information on age, marital status, height, weight, ancestry, medications, smoking history, medical history, physical activity, and diet was collected, and medical history information is updated every two years. Blood samples were collected from 18,225 of the HPFS participants from 1993 to1995. For the present study, we used 1,245 control individuals with GWAS and serum 25(OH)D data taken from a case-control study of coronary heart disease in the HPFS [[10](#_ENREF_10)].

*Measurement of serum 25(OH)D*: Plasma samples were measured by radioimmunoassay (RIA) in Dr. Hollis’ lab (CV: 11.5%).

*Measurement of BMI*: The HPFS mailed questionnaire includes items about their medical history including height and weight. These measures taken at the time of blood draw were used to compute BMI defined as weight in kilograms divided by height in meters to the second power. Should these measures not be available at the time of blood draw, the information was taken from the preceding questionnaire (2 years prior).

*Genotyping*: GWAS genotyping used the Affymetrix 6.0 platform in the T2D. Genotypes for markers contained on the Illumina 550K platform and not the Affymetrix 6.0 platform were imputed using a Markov Chain based Haplotyper (MACH) and HapMap CEU reference panel (Release 22), with retention of only high quality SNPs with a MACH-r2 of 0.95 or greater in all five data sets. Quality-control assessment of genotypes including sample completion rates, SNP call rates, concordance rates, deviation from Hardy–Weinberg proportions in control DNA and final sample selection for associational analysis are described in detail elsewhere.

1. **Case-control study of type II diabetes (NHS-T2D)**

*Data collection*: Nurses’ Health Study (NHS) began in 1976 when 121,700 female US registered nurses, 30 to 55 years of age were enrolled. The cohort is contacted every 2 years by questionnaire to update diet, medications, anthropometrics and incident physician-diagnosed illnesses with response rates to the follow-up questionnaires are >90% in each cycle. Between 1989 and 1990, 32,826 women between the ages of 43 and 69, have provided blood samples. Cases were matched to non-diabetic controls on age, month, and year of blood draw and fasting status. To minimize potential bias due to population stratification, we restricted our analyses to non-Hispanic whites. 720 women with GWAS data and serum 25(OH)D were taken from a nested case-control study of type II diabetes (T2D) [[11](#_ENREF_11)] and were used in the present study.

*Measurement of serum 25(OH)D*: Plasma levels of 25(OH)D were measured in the Nutrition Evaluation Laboratory in the Human Nutrition Research Center on Aging at Tufts University (CV 8.7%) by rapid extraction followed by equilibrium I-125 RIA procedure as specified by the manufacturer’s procedural documentation and analyzed by gamma counter (Cobra II, Packard).

*Measurement of BMI*: The NHS mailed questionnaire includes items about their medical history including height and weight. These measures taken at the time of blood draw were used to compute BMI defined as weight in kilograms divided by height in meters to the second power. Should these measures not be available at the time of blood draw, the information was taken from the preceding questionnaire (2 years prior).

*Genotyping*: GWAS genotyping used the Affymetrix 6.0 platform in the T2D. Genotypes for markers contained on the Illumina 550K platform and not the Affymetrix 6.0 platform were imputed using a Markov Chain based Haplotyper (MACH) and HapMap CEU reference panel (Release 22), with retention of only high quality SNPs with a MACH-r2 of 0.95 or greater in all five data sets. Quality-control assessment of genotypes including sample completion rates, SNP call rates, concordance rates, deviation from Hardy-Weinberg proportions in control DNA and final sample selection for associational analysis are described in detail elsewhere.

1. **Framingham Heart Study (FHS):**

*Data collection*: The FHS consists of three cohorts. The original cohort was recruited in 1948 and includes 5,209 participants from Framingham, Massachusetts, US [[12](#_ENREF_12)]. Clinical exams were conducted every other year to investigate cardiovascular disease and related risk factors. The Offspring cohort is comprised of 5,124 children of the Original cohort and the children's spouses. They were recruited in 1971, and have undergone examinations roughly every four years. A total 4,095 children of the Offspring cohort form the Third Generation cohort were enrolled in 2002. The current study sample consists of 1,815 individuals from the Offspring Generation who attended the 5th examination (1991-1995) and 3,841 from the Third Generation who attended the 1st examination (2002-2005).

*Measurement of serum 25(OH)D*: 25(OH)D was determined by a competitive protein-binding assay. Intra-assay CV for this assay was 7%.

*Measurement of BMI*: BMI was derived as weight (in kilograms) divided by the square of height (in meters).

*Genotyping*: Genotyping was performed using the Affymetrix 500K SNP arrays supplemented with the MIPS 50K array. Samples were excluded if they had call rate < 97%, excess Mendelian errors (> 1000) or average heterozygosity outside of 5 SD of mean (< 25.758% or > 29.958%).

1. **Health, Aging and Body Composition (Health ABC) Study:**

*Data collection*: The Health ABC study is a prospective cohort study investigating the associations between body composition, weight-related health conditions, and incident functional limitation in older adults [[13](#_ENREF_13)]. Health ABC enrolled well-functioning, community-dwelling black (n=1,281) and white (n=1,794) men and women aged 70-79 years between April 1997 and June 1998. Participants were recruited from a random sample of white and all black Medicare eligible residents in the Pittsburgh, PA, and Memphis, TN, metropolitan areas in the United States. Participants have undergone annual exams and semi-annual phone interviews. The current study sample consists of 1,558 white participants who attended the second exam in 1998-1999 with available genotyping and serum 25(OH)D concentrations.

*Measurement of serum 25(OH)D*: Serum 25(OH)D concentrations were measured using fasting blood samples collected in the morning after a 12-hour fast, centrifuged, and stored at -80° C. Serum 25(OH)D was measured using a 2-step radioimmunoassay (25-Hydroxyvitamin D 125I RIA Kit, DiaSorin, Stillwater, Minn., USA). The inter- and intra-assay coefficients of variation for serum 25(OH)D were 6.78% and 9.16%, respectively.

*Measurement of BMI*: Weight without shoes or heavy jewellery and wearing a standard clinic gown was measured in kilograms using a standard balance beam scale. Height without shoes was measured in millimetres using a Harpenden stadiometer (Holtain Ltd., Crosswell, United Kingdom). Body mass index was calculated as weight (in kg) / height (in m) squared.

*Genotyping*: Genotyping was performed by the Center for Inherited Disease Research (CIDR) using the Illumina Human1M-Duo BeadChip system. Samples were excluded from the dataset for the reasons of sample failure, genotypic sex mismatch, and first-degree relative of an included individual based on genotype data. Genotyping was successful in 1663 Caucasians. Analysis was restricted to SNPs with minor allele frequency ≥ 1%, call rate ≥97% and HWE p≥10-6. Genotypes were available on 914,263 high quality SNPs for imputation based on the HapMap CEU (release 22, build 36) using the MACH software (version 1.0.16).

1. **Health2000 GenMets (GENMETS) Study:**

*Data collection*: The GENMETS sample is a subset of 2,212 individuals of the Health2000 study [[14](#_ENREF_14)] collected as metabolic syndrome cases and their matched controls. The health 2000 survey was conducted in 2000 and 2001 in Finland. The survey included an interview on medical history, health-related lifestyle habits, and a

clinical examination where a blood sample was drawn. For the present study, a total of 868 controls with genotype data, BMI and vitamin D measurements were included.

*Measurement of serum 25(OH)D*: Serum 25(OH)D concentrations were measured using 25-hydroxyvitamin D RIA kit method (DiaSorin, Stillwater, MN, USA). The intra-assay coefficient of variation (CV) was 3,5 % and the interassay CV was 6.9 % at the concentration of 36 nmol/l. The limit of detection was 3,5 nmol/l. The serum specimens were stored frozen at - 70 C until analysed and protected from light when processed.

*Measurement of BMI*: BMI was calculated as weight (kg)/height (m^2^) using height measured with wall-mounted stadiometer and weight measured with digital scale or bioimpedance device.

*Genotyping*: Genotyping was done on Illumina 610K arrays. 598,203 SNPs were successfully called with Illuminus software. Sample exclusions included sample call rate (<95%), relatedness, heterozygosity and gender discrepancy. The SNP exclusions were done for MAF < 1%, call rate < 95%, SNP clustering probability for each genotype < 95%, HWE p < 1x10^-6^.

1. **Hertfordshire cohort study (HCS)**

*Data collection*: The Hertfordshire Cohort Study (HCS) has recruited 3000 men and women born in Hertfordshire UK during 1931-9 and still living there in adult life [[15](#_ENREF_15)]. Participants in the study have been followed regularly with information collected on a wide range of factors related to health, lifestyle and infant growth. During 1998-2003 cohort members attended a research clinic, which included anthropometric data collection and blood sampling for genetic studies and serum 25(OH)D concentration. Full informed consent was obtained. In total, the present study included 2,997 individuals.

*Measurement of serum 25(OH)D*: Automated DiaSorin chemiluminescent assay (CV 10-12% within batch, and 10-15% between batch) was used to measure 25(OH)D.

*Measurement of BMI*: Height was measured to the nearest 0.1cm using a Harpenden pocket stadiometer; weight was measured to the nearest 0.1kg on a SECA floor scale. *Genotyping*: Genotyping was performed using TaqMan SNP genotyping assays (Applied Biosystems, Warrington, UK) according to the manufacturer’s protocol. Genotype frequencies were in HWE (p>0.01). Call rates were >98%. 100% concordances of duplicates (n=300) were observed.

1. **InCHIANTI study**

*Data collection*: The InCHIANTI study comprises of over 1200 aging individuals from Chianti, Tuscany in the northern region of Italy [[16](#_ENREF_16)]. Participants in the study have been followed up every 3 years in order to record information regarding health and lifestyle. During the first wave (1998) participants undertook a medical examination in which blood measures were taken in addition to anthropometric traits. Total of 1,210 individuals had all necessary covariates available and were analysed in the present study.

*Measurement of serum 25(OH)D*: Serum levels of vitamin D (25OHD) were measured by radioimmunoassay (RIA kit; DiaSorin, Stillwater, MN). The intra-assay and inter-assay coefficients of variation for vitamin D were 8.1% and 10.2%, respectively.

*Measurement of BMI*: Height and weight were measured at the study clinic, and BMI was calculated as weight (in kg) divided by the square of height (in m).

*Genotyping*: The samples were genotyped using the Illumina Infinium HumanHap550 platform. Samples were initially assessed for genotype success rate (>98%) and concordance of reported and genotype gender. Nine samples were removed from further analysis due to gender mismatch. Eighty seven samples failed the cut off genotype success rate of 98%; forty eight of these samples were re-purified and successfully genotyped, thus in total 48 samples were removed from further analysis.

1. **Ludwigshafen Risk and Cardiovascular Health (LURIC) Study:**

*Data collection*: The LURIC Study is a prospective cohort study among 3,316 study participants who were routinely referred to a tertiary care medical centre in south-west Germany between 1997 and 2000 [[17](#_ENREF_17)]. Inclusion criteria were the availability of a coronary angiogram, German ancestry and clinical stability with the exception of acute coronary syndromes (ACS). Exclusion criteria were any acute illness other than ACS, any chronic disease where non-cardiac disease predominated and a history of malignancy within the past five years. Patients were continuously followed up with respect to fatal events. 25(OH)D was measured in 3,299 patients of the LURIC study.

*Measurement of serum 25(OH)D*: 25(OH)D was measured in serum by means of a radioimmunoassy (RIA) (DiaSorin, Antony, France, and Stillwater, MN) with intra- and inter-assay coefficients of variation of 8.6 and 9.2, respectively.

*Measurement of BMI*: Body weight was measured without shoes and in light clothing by a trained nurse. Body height was recorded to the nearest centimetre with the subject barefoot and in the upright position. Body mass index was calculated as weight (kg)/hight (m)². Body mass index was available in all 3,316 study participants.

*Genotyping*: DNA samples of 1,985 patients were genotypes with the Affymetrix Genome-Wide Human SNP Array 6.0 (Affymetrix 6.0) and 500K. Samples with low call rate (<90 %), cryptic relatedness or gender discrepancy were excluded from analyses. SNP exclusions were done for MAF<1%, HWE <10e^-6^ and low call rate (<95%).

1. **Medical Research Council Ely (MRC Ely) Study:**

*Data collection*: The participants in this study are a subset of the Ely Study (Cambridgeshire, U.K.), which was established in 1990 as a prospective study; detailed methodology has been described previously [[18](#_ENREF_18)]. In brief, from a sampling frame of all adults free of known diabetes and registered with a single practice serving Ely, 1,122 European-origin adults, aged 40–69 years, were randomly selected (response rate 74%). The baseline examination, during which blood samples were taken for measurement of serum 25(OH)D concentrations, took place between 1990 and 1992. Blood samples for DNA extraction were obtained during the second follow-up examination that took place between 2000-2003. Of the 1,122 individuals, 337 individuals were excluded from analyses because of missing data for serum 25(OH)D concentrations (n=5) and missing DNA due to lost to follow-up (n = 332). A total of 785 individuals formed the present study population.

*Measurement of serum 25(OH)D*: Fasting serum 25(OH)D concentration was measured by radioimmunoassay using acetonitrile extracts of serum [[19](#_ENREF_19)] (CV <15%).

*Measurement of BMI*: Participants attended health check examinations in the morning after an overnight fast. We performed anthropometric measurements to standard protocol, including weight (kg), and height (m), and calculated BMI (weight in kilograms divided by the square of height in meters [kg/m^2^].

*Genotyping*: Most SNPs were genotyped using the 'metabochip' which is a 200k bead‐type custom array from Illumina. A few SNPs were genotyped on the Sequenom iPLEX system, whereas the two vitD SNPs were genotyped using TaqMan. The call rate and the concordance rate for genotyping ranged from 95-100% and 97-100%, respectively.

1. **Northern Finland Birth Cohort 1966 (NFBC1966) study**

*Data collection*: NFBC1966 was originally designed to study factors affecting pre‐term birth, low birth weight and subsequent morbidity and mortality [[20](#_ENREF_20)]. Mothers living in the two northern‐most provinces of Finland were invited to participate if they had expected delivery dates during 1966. A total of 12,058 live‐births were in the study. At age 31 all individuals still living in the Helsinki area or Northern Finland were asked to participate in a detailed biological and medical examination (n=6,007) as well as a questionnaire. Genotype and measured BMI data were available on 4,453 individuals in this study with multiple births being excluded.

*Measurement of serum 25(OH)D*: Total 25-hydroxyvitamin D was measured with high-performance liquid chromatography-tandem mass spectrometry.

*Measurement of BMI*: Anthropometric measures, including height, weight and waist circumference were taken.

*Genotyping*: For NFBC, genomic DNA was extracted from whole blood using standard methods. All DNA samples for the Illumina Infinium 370cnvDuo array were prepared for genotyping by the Broad Institute Biological Sample Repository (BSP). All individuals in the study were genotyped with call rates >95%. SNPs were excluded from analysis if the call rate in the final sample was < 95%, if the *P* value from a test of HWE was <0.0001, or if the MAF was <1%.

1. **Prospective Investigation of the Vasculature in Uppsala Seniors (PIVUS):**

*Data collection*: The PIVUS (http://www.medsci.uu.se/pivus/pivus.htm) has been described in detail previously [[21](#_ENREF_21)]. Briefly, all 70-yr-old individuals living in Uppsala, Sweden, in 2001–2004 were eligible for the PIVUS, and 2025 randomly selected individuals were invited within 2 months of their 70th birthday from April 2001 to June 2004. Of these, 1016 (50%) participated in the study. At the examination the participants underwent a blood pressure measurement and anthropometry, blood sampling after an overnight fast, routine medical history, and assessment of BMD using dual-energy x-ray absorptiometry (DXA). In total, the present study included 1,013 individuals.

*Measurement of serum 25(OH)D*: 25-hydroxyvitamin D2 and D3 in serum were measured as a standard procedure at the department of Clinical Chemistry at Uppsala University Hospital. The LIAISON® 25-hydroxyvitamin D Assay (DiaSorin) uses chemiluminescent immunoassay technology. CV for inter-assay analyses is 18.4% at a 25-hydroxyvitamin D level of 39.5 nmol/L and 11.7% at 121.2 nmol/L. The intra-assay CV is 7.1% at 44.7 nmol/L and 3.6% at 120.0 nmol/L.

*Measurement of BMI*: Weight and standing height (n=1194) were measured without shoes and in light clothing by a trained nurse. Body mass index was calculated as weight (kg)/height (m)^2^.

*Genotyping*: The SNPs were genotyped as part of a larger panels of SNPs at the SNP technology platform at Uppsala University (http://www.genotyping.se/) on an Illumina BeadStation 500GX using Infinium iSelect and Golden Gate assays from Illumina Inc [[22](#_ENREF_22)]. Genotyping calls were performed with Illumina BeadStudio or GenCall software. Samples with low call rate (<90 %), excess heterozygosity or cryptic relatedness were excluded from analyses. SNPs with a call rate less than 90%, or that failed HWE (exact P-value<1x10^-6^) were excluded from the study.

1. **Scottish Colorectal Cancer Study (SOCCS):**

*Data collection*: SOCCS is the Scottish national case control study of colorectal cancer (the Study of Colorectal Cancer in Scotland, SOCCS) [[23](#_ENREF_23)]. Participants were recruited in the period from 1999 to 2006. Wide range of lifestyle, diet and health information was collected, including measurements of vitamin D. The whole cohort includes 4,284 participants, but only ~2000 were genotyped. Total of 1,176 controls had all necessary covariates available and were analysed in the present study.

*Measurement of serum 25(OH)D*: The liquid chromatography-tandem mass spectrometry (LC-MS/MS) method was used to measure 25-hydroxyvitamin D3 and D2. The LC-MS/MS method was performed following standard protocols and appropriate quality control procedures (including standardisation against standard reference material, SRM 972). CV for inter- and intra-assay analyses for 25-hydroxyvitamin D3 and D2 is less than 10% [[24](#_ENREF_24)].

*Measurement of BMI*: BMI was calculated from self-reported height and weight information.

*Genotyping*: 2000 DNA samples were genotyped, but for only ~1,600, vitamin D measurements were available. Samples were genotyped on the Illumina Hum300 and 240S, and genotype calling algorithm was Beadstudio. Sample exclusions included exceeding heterozygosity thresholds, ethnic outliers, gender discrepancy and low call rate (less than 90%). SNP exclusions were done for MAF<1%, HWE <10e^-6^ and low call rate (<95%).

1. **The Gothenburg Osteoporosis and Obesity Determinants (GOOD) Study:**

*Data collection*: The Gothenburg Osteoporosis and Obesity Determinants (GOOD) study was initiated to determine both environmental and genetic factors involved in the regulation of bone and fat mass [[25](#_ENREF_25)]. Male study participants were randomly identified in the greater Gothenburg area in Sweden using national population registers, contacted by telephone, and invited to participate. To be enrolled in the GOOD study, participants had to be between 18 and 20 years of age. There were no other exclusion criteria, and 49% of the study candidates agreed to participate. A total of 921 individuals were included for the present study.

*Measurement of serum 25(OH)D*: Serum levels of 25-hydroxyvitamin D were measured by radioimmunoassay (DiaSorin Inc, Stillwater, MN) (n = 921). Intra-assay CV was 6%.

*Measurement of BMI*: Height was measured using a wall-mounted stadiometer, and weight was measured to the nearest 0.1 kg. Body mass index was calculated as weight (kg)/height (m^2^).

*Genotyping*: Genotyping was performed with Illumina HumanHap610 arrays at the Genetic Laboratory, Department of Internal Medicine, Erasmus Medical Center, Rotterdam, the Netherlands. Genotypes were called using the BeadStudio calling algorithm. Genotypes from 938 individuals passed the sample quality control criteria [exclusion criteria: sample call rate <97.5%, gender discrepancy with genetic data from X-linked markers, excess autosomal heterozygosity >0.33 (~FDR < 0.1%), duplicates and/or first degree relatives identified using IBS probabilities (>97%), ethnic outliers (3 SD away from the population mean) using multi-dimensional scaling analysis with four principal components]. We sequentially discarded intensity-only markers and markers with a SNP call rate <95%.

1. **Twins UK**

*Data collection*: The Twins registry in St. Thomas' Hospital, King's College London recruited a total sample of 11,000 identical and non-identical, mostly female Caucasian, twins from across the UK through national media campaigns [[26](#_ENREF_26)]. Their age ranges between 16 and 85 years. Over 7,000 twins have attended detailed clinical examinations with a wide range of phenotypes over last 18 years. All participants were recruited without presence or interest in any particular disease or trait. We used 1,930 individuals for the present study.

*Measurement of serum 25(OH)D*: Total 25-hydroxyvitamin D levels were measured by radioimmunoassay using Diasorin RIA kit (Diasorin, Minnesota, USA). This assay has a detection limit of 4 nmol/L and CV of 9.1% at 22 nmol/L.

*Measurement of BMI*: Weight and standing height were measured without shoes and in light clothing by a trained nurse. Body mass index was calculated as weight (kg)/height (m)^2^.

*Genotyping*: Genotyping in the TwinsUK study was performed using Illumina 610K SNP. Analyses were restricted to SNPs with call rate of genotype ≥ 98% with average heterozygosity within 3 SD of mean (< 0.3042438 and > 0.3267991). MAF was set as >1% and HWE P > 10-4. IMPUTE software was used for imputation.

1. **UK Blood Services Common Control Collection (UKBS-CC)**

*Data collection*: The UKBS-CC is an anonymised collection of 3100 DNA samples from healthy blood donors (mean age: 45 years). The collection has been established by the three British blood services of England, Scotland, and Wales as part of the Wellcome Trust Case Control Consortium (WTCCC) study [[27](#_ENREF_27)] (<http://www.wtccc.org.uk/>).  For the present study, we included 2,638 individuals with genotype, vitamin D and BMI measurements.

*Measurement of serum 25(OH)D*: 25(OH)D was measured in serum by means of a radioimmunoassy (RIA) (DiaSorin). The inter- and intra-assay CVs were 6.3% and 2.2%, respectively.

*Measurement of BMI*: Self-reported height and weight was collected at time of consent.

*Genotyping*: 3,000 DNA samples were selected as control subjects in the Welcome Trust Case Control Consortium 2 (WTCCC2). This includes an initial 1,500 sampled in the first round of WTCCC. The samples were genotyped on the Affymetrix 6.0 and Illumina 1.2M platforms.

1. **Uppsala Longitudinal Study of Adult Men (ULSAM)**

*Data collection*: The ULSAM was initiated in 1970 when all men born during the years 1920-24 and living in Uppsala, Sweden were invited at age 50 to participate in a health survey focusing on identifying cardiovascular risk factors (<http://www.pubcare.uu.se/ULSAM>) [[28](#_ENREF_28)]. The present analyses are based on measurements made at the third examination cycle of the ULSAM cohort, when participants were approximately 71 years old (1991-95, n=1221). Of these, 1,194 participants had measurements of plasma 25(OH)D and were included in this study.

*Measurement of serum 25(OH)D*: Total plasma 25(OH)D, including 25-hydroxyvitamin D3 and D2, was determined with high-pressure liquid chromatography (HPLC) atmospheric pressure chemical ionization (APCI) mass spectrometry (MS) at Vitas, Oslo, Norway ([www.vitas.no](http://www.vitas.no)). HPLC was performed with a Hewlett Packard 1100 liquid chromatography (Agilent Technologies, Palo Alto CA, USA) interfaced by APCI to a Hewlett Packard mass spectrometer operated in single-ion monitoring mode (SIM). Recovery is 95%; the method is linear from 5-400 nmol/L and the limit of detection is 1-4 nmol/L. The Coefficients of Variation (CV) for inter-assay analyses are 7.6% at 25-hydroxyvitamin D concentrations of 47.8 nmol/L and 6.9% at 83.0 nmol/L. The intra-assay CV was 5.1% at 47.8 nmol/L and 6.1% at 83.0 nmol/L. The assay is accredited by the Vitamin D External Quality Assessment Scheme (DEQAS).

*Measurement of BMI*: Weight and standing height (n= 1,194) were measured without shoes and in light clothing by a trained nurse. Body mass index was calculated as weight (kg)/height (m)^2^.

*Genotyping*: The SNPs were genotyped as part of a larger panels of SNPs at the SNP technology platform at Uppsala University (<http://www.genotyping.se/>) on an Illumina BeadStation 500GX using Infinium iSelect and Golden Gate assays from Illumina Inc.. Genotyping calls were performed with Illumina BeadStudio or GenCall software. Samples with low call rate (<90 %), excess heterozygosity or cryptic relatedness were excluded from analyses. SNPs with a call rate less than 90%, or that failed (exact P-value<1x10^-6^) were excluded from the study.

1. **Young Finns Study:**

*Data collection*: The first cross-sectional survey was conducted in 1980 all around Finland [[29](#_ENREF_29)]. Total sample size was 4,320 boys and girls in 6 age cohorts (aged 3, 6, 9, 12, 15 and 18). These individuals were randomly chosen from the national register. A total of 3,596 individuals (83.2% of those invited) participated the study in 1980. After that, several follow-up studies of this cohort have been conducted. The latest follow-up was performed in 2007, when the study subjects had reached the age of 30 to 45 years. In the latest follow-up in 2007 a total of 2,204 subjects were examined [[30](#_ENREF_30)]. For the present study, we included 2,442 individuals with genotype, BMI and vitamin D measurements.

*Measurement of serum 25(OH)D*: Serum 25(OH)D concentrations were determined using radioimmunoassay (DiaSorin, Stillwater, Minnesota). The detection limit was 3.8 nmol/l, sensitivity = 1 nmol/l, measurement range = 12.5 - 250 nmol/l, inter-assay CV 8.0% (n = 137) and intra-assay CV 1.8 – 11.0% (n = 5) at the mean level of 43.8 nmol/l).

*Measurement of BMI*: Weight and standing height (n=2170) were measured without shoes and in light clothing by a trained nurse. Body mass index was calculated as weight (kg)/height (m)^2^.

*Genotyping*: The genome-wide SNP genotyping of YF-study was done by a custom Illumina BeadChip containing 670,000 SNPs and copy-number variant (CNV) probes from 2,442 YF participants (1,123 males, 1,319 females). The custom 670K chip shares 562,643 SNPs in common with the Illumina Human610 BeadChip. Genotypes were called using Illumina’s clustering algorithm (Illuminus). A total of 2,556 samples were genotyped. After initial clustering, we removed 2 individuals for poor call rates (CR<0.90), and 54 samples failed subsequent quality control (QC) (i.e., duplicated samples, heterozygosity, low call rate, or custom SNP fingerprint genotype discrepancy). The following filters were applied to the remaining data: MAF 0.01, GENO 0.05, MIND 0.05, and HWE 1 x 10-6. Three of 2,500 individuals were removed for low genotyping (MIND > 0.05), 11,766 markers were excluded based on HWE test (P ≤ 1 x 10-6), 7,746 SNPs failed missingness test (GENO > 0.05), 34,596 SNPs failed frequency test (MAF < 0.01) and one individual failed gender check. None were removed by subsequent heterozygosity check. In that point, there were 546,770 SNPs and 2,496 individuals which were utilized to generate an identity-by-descent (IBD) matrix file in PLINK. There were 51 pairs of individuals with pi-hat greater than 0.2 thus these individuals removed due to possible relatedness. One of the pair was removed using greater missingness as criteria. After final frequency and genotyping running, there was 546,677 SNPs available from a sample of 2442 Young Finns individuals. Genotype imputation was performed for the YF SNP data using MACH with the HapMap (phase II, release 22 CEU, NCBI build 36, dbSNP 126) haplotypes as reference.

**References**

1. Power C, Elliott J (2006) Cohort profile: 1958 British birth cohort (National Child Development Study). Int J Epidemiol 35: 34-41.

2. Craddock N, Hurles ME, Cardin N, Pearson RD, Plagnol V, et al. (2010) Genome-wide association study of CNVs in 16,000 cases of eight common diseases and 3,000 shared controls. Nature 464: 713-720.

3. Todd JA, Walker NM, Cooper JD, Smyth DJ, Downes K, et al. (2007) Robust associations of four new chromosome regions from genome-wide analyses of type 1 diabetes. Nat Genet 39: 857-864.

4. Brown LB, Streeten EA, Shuldiner AR, Almasy LA, Peyser PA, et al. (2004) Assessment of sex-specific genetic and environmental effects on bone mineral density. Genet Epidemiol 27: 153-161.

5. Kreiger N, Tenenhouse, A., Joseph, L., Mackenzie, T., Poliquin, S., Brown, J.P., Prior, J.C., Rittmaster, R.S. (1999) The Canadian Multicentre Osteoporosis Study (CaMos): Background, Rationale, Methods. Can J Aging 18: 376-387.

6. Colditz GA, Martin P, Stampfer MJ, Willett WC, Sampson L, et al. (1986) Validation of questionnaire information on risk factors and disease outcomes in a prospective cohort study of women. Am J Epidemiol 123: 894-900.

7. Hollis BW (1997) Quantitation of 25-hydroxyvitamin D and 1,25-dihydroxyvitamin D by radioimmunoassay using radioiodinated tracers. Methods Enzymol 282: 174-186.

8. Bertone-Johnson ER, Chen WY, Holick MF, Hollis BW, Colditz GA, et al. (2005) Plasma 25-hydroxyvitamin D and 1,25-dihydroxyvitamin D and risk of breast cancer. Cancer Epidemiol Biomarkers Prev 14: 1991-1997.

9. Rimm EB, Giovannucci EL, Stampfer MJ, Colditz GA, Litin LB, et al. (1992) Reproducibility and validity of an expanded self-administered semiquantitative food frequency questionnaire among male health professionals. Am J Epidemiol 135: 1114-1126; discussion 1127-1136.

10. Giovannucci E, Liu Y, Hollis BW, Rimm EB (2008) 25-hydroxyvitamin D and risk of myocardial infarction in men: a prospective study. Arch Intern Med 168: 1174-1180.

11. Cornelis MC, Qi L, Kraft P, Hu FB (2009) TCF7L2, dietary carbohydrate, and risk of type 2 diabetes in US women. Am J Clin Nutr 89: 1256-1262.

12. Jaquish CE (2007) The Framingham Heart Study, on its way to becoming the gold standard for Cardiovascular Genetic Epidemiology? BMC Med Genet 8: 63.

13. Shea MK, Houston DK, Tooze JA, Davis CC, Johnson MA, et al. (2011) Correlates and prevalence of insufficient 25-hydroxyvitamin D status in black and white older adults: the health, aging and body composition study. J Am Geriatr Soc 59: 1165-1174.

14. Methodology Report: Health 2000 Survey. . <http://wwwterveys2000fi/doc/methodologyreppdf>.

15. Dennison EM, Syddall HE, Aihie Sayer A, Martin HJ, Cooper C (2007) Lipid profile, obesity and bone mineral density: the Hertfordshire Cohort Study. Qjm 100: 297-303.

16. Melzer D, Perry JR, Hernandez D, Corsi AM, Stevens K, et al. (2008) A genome-wide association study identifies protein quantitative trait loci (pQTLs). PLoS Genet 4: e1000072.

17. Winkelmann BR, Marz W, Boehm BO, Zotz R, Hager J, et al. (2001) Rationale and design of the LURIC study--a resource for functional genomics, pharmacogenomics and long-term prognosis of cardiovascular disease. Pharmacogenomics 2: S1-73.

18. Wareham NJ, Byrne CD, Williams R, Day NE, Hales CN (1999) Fasting proinsulin concentrations predict the development of type 2 diabetes. Diabetes Care 22: 262-270.

19. Forouhi NG, Luan J, Cooper A, Boucher BJ, Wareham NJ (2008) Baseline serum 25-hydroxy vitamin d is predictive of future glycemic status and insulin resistance: the Medical Research Council Ely Prospective Study 1990-2000. Diabetes 57: 2619-2625.

20. Jarvelin MR, Sovio U, King V, Lauren L, Xu B, et al. (2004) Early life factors and blood pressure at age 31 years in the 1966 northern Finland birth cohort. Hypertension 44: 838-846.

21. Lind L, Fors N, Hall J, Marttala K, Stenborg A (2005) A comparison of three different methods to evaluate endothelium-dependent vasodilation in the elderly: the Prospective Investigation of the Vasculature in Uppsala Seniors (PIVUS) study. Arterioscler Thromb Vasc Biol 25: 2368-2375.

22. Fan JB, Oliphant A, Shen R, Kermani BG, Garcia F, et al. (2003) Highly parallel SNP genotyping. Cold Spring Harb Symp Quant Biol 68: 69-78.

23. Theodoratou E, Kyle J, Cetnarskyj R, Farrington SM, Tenesa A, et al. (2007) Dietary flavonoids and the risk of colorectal cancer. Cancer Epidemiol Biomarkers Prev 16: 684-693.

24. Knox S, Harris J, Calton L, Wallace AM (2009) A simple automated solid-phase extraction procedure for measurement of 25-hydroxyvitamin D3 and D2 by liquid chromatography-tandem mass spectrometry. Ann Clin Biochem 46: 226-230.

25. Lorentzon M, Swanson C, Andersson N, Mellstrom D, Ohlsson C (2005) Free testosterone is a positive, whereas free estradiol is a negative, predictor of cortical bone size in young Swedish men: the GOOD study. J Bone Miner Res 20: 1334-1341.

26. Spector TD, Williams FM (2006) The UK Adult Twin Registry (TwinsUK). Twin Res Hum Genet 9: 899-906.

27. (2007) Genome-wide association study of 14,000 cases of seven common diseases and 3,000 shared controls. Nature 447: 661-678.

28. Michaelsson K, Baron JA, Snellman G, Gedeborg R, Byberg L, et al. (2010) Plasma vitamin D and mortality in older men: a community-based prospective cohort study. Am J Clin Nutr 92: 841-848.

29. Kivimaki M, Magnussen CG, Juonala M, Kahonen M, Kettunen J, et al. (2011) Conventional and Mendelian randomization analyses suggest no association between lipoprotein(a) and early atherosclerosis: the Young Finns Study. Int J Epidemiol 40: 470-478.

30. Raitakari OT, Juonala M, Ronnemaa T, Keltikangas-Jarvinen L, Rasanen L, et al. (2008) Cohort profile: the cardiovascular risk in Young Finns Study. Int J Epidemiol 37: 1220-1226.

**Funding**

**1958 Birth cohort (1958BC):** The 25-hydroxyvitamin D assays were funded by the BUPA foundation. DNA collection was funded by MRC grant G0000934 and cell-line creation by Wellcome Trust grant 068545/Z/02. This research used resources provided by the Type 1 Diabetes Genetics Consortium, a collaborative clinical study sponsored by the National Institute of Diabetes and Digestive and Kidney Diseases (NIDDK), National Institute of Allergy and Infectious Diseases, National Human Genome Research Institute, National Institute of Child Health and Human Development, and Juvenile Diabetes Research Foundation International (JDRF) and supported by U01 DK062418. This study makes use of data generated by the Wellcome Trust Case-Control Consortium. A full list of investigators who contributed to generation of the data is available from the Wellcome Trust Case-Control Consortium website. Funding for the project was provided by the Wellcome Trust under award 076113. Great Ormond Street Hospital/University College London, Institute of Child Health and Oxford Biomedical Research Centre, University of Oxford receive a proportion of funding from the Department of Health's National Institute for Health Research (NIHR) ('Biomedical Research Centres' funding). This paper presents independent research and the views expressed are those of the author(s) and not necessarily those of the NHS, the NIHR, or the Department of Health.

**Amish Family Osteoporosis Study (AFOS):** The Amish Family Osteoporosis Study was funded by support from NIH research grants R01 AR46838, F32 AR059469 and R01_HL088119. Partial funding was provided by the Mid-Atlantic Nutrition and Obesity Research Center of Maryland (P30DK072488).

**Canadian Multicentre Osteoporosis Study (CaMos):** Canadian Multicentre Osteoporosis Study is funded by the Canadian Institutes of Health Research (CIHR). JBR is a clinical investigator of the CIHR and an Osteoporosis Canada New Investigator. This work was supported by grants to JBR from the CIHR and Canadian Foundation for Innovation. We acknowledge the support and funding of the Public Health Agency of Canada to assay vitamin D levels, the expert technical support of Kurtis Sarafi and Jenn Kreiger of Health Canada (vitamin D measurements) and the input of Linda S Greene-Finestone and Ross Duncan.

**Cancer Genetic Markers of Susceptibility, case control study of breast cancer (NHS-CGEMS)**: David Hunter, the Principal Investigator for the study of breast cancer in the Nurses Health Study; Carolyn Guo, Programmer.

**Case-control study of coronary heart disease (HPFS-CHD)**: Eric Rimm, the Principal Investigator for the study of coronary heart disease in the Health Professionals Follow-Up Study; Carolyn Guo, Programmer.

**Case-control study of type II diabetes (NHS-T2D)**: Frank Hu, the Principal Investigator for the study of type II diabetes in the Nurses Health Study; Carolyn Guo, Programmer.

**Framingham Heart Study** (**FHS)**: The Framingham Heart Study of the National Heart, Lung and Blood Institute of the National Institutes of Health and Boston University School of Medicine is supported by the NIH/NHLBI contract N01-HC-25195. The present study received support from the American Heart Association, the U.S. Department of Agriculture, Agricultural Research Service (under Cooperative Agreement No. 58-1950-7-707), and the National Institute of Aging (AG14759). Dr. Kiel was supported by a grant from the National Institute of Arthritis, Musculoskeletal, and Skin Diseases and the National Institute on Aging (R01 AR/AG 41398). The analyses reflect intellectual input and resource development from the Framingham Heart Study investigators participating in the SNP Health Association Resource (SHARe) project. This work was partially supported by a contract with Affymetrix, Inc for genotyping services (Contract No. N02-HL-6-4278). A portion of this research utilized the Linux Cluster for Genetic Analysis (LinGA-II) funded by the Robert Dawson Evans Endowment of the Department of Medicine at Boston University School of Medicine and Boston Medical Center.

**Health, Aging and Body Composition (Health ABC) Study:** The Health ABC study was supported by the Intramural Research Program of the National Institutes of Health, National Institute on Aging and National Institute on Aging contracts N01-AG-6-2101, N01-AG-6-2103, and N01-AG-6-2106. Assessment of 25-hydroxyvitamin D concentrations was funded by a National Institute on Aging grant, R01-AG029364. The genome-wide association study was funded by a National Institute on Aging grant, R01-AG032098, and genotyping services were provided by the Center for Inherited Disease Research (CIDR). CIDR is fully funded through a federal contract from the National Institutes of Health to The Johns Hopkins University (contract number HHSN268200782096C). We also thank Yongmei Liu (Co-PI of Health ABC GWAS grant), who obtained GWAS data, reviewed analysis proposal and the manuscript draft.

**Health2000 GenMets (GENMETS) Study:** The authors would like to thank the many colleagues who contributed to the phenotypic characterization of GENMETS participants, collection of clinical data and DNA extraction and genotyping, especially Eija Hämäläinen, Minttu Jussila, Outi Törnwall, Päivi Laiho, and the staff from the genotyping facilities at the Wellcome Trust Sanger Institute. They would also like to acknowledge those who agreed to participate in this study. Dr Salomaa was supported by the Academy of Finland (grant numbers 129494 and 139635) and the Finnish Foundation for Cardiovascular Research.

**Hertfordshire cohort study (HCS):** Genotyping was in part funded by the British Heart Foundation (grant PG/09/023). The HCS is supported by the Medical Research Council of Great Britain; Arthritis Research, UK; the British Heart Foundation; Porticus Foundation; the International Osteoporosis Foundation and the NIHR Biomedical Research Unit in Nutrition, University of Southampton, UK.

**InCHIANTI study**: The InCHIANTI study was supported by contract funding from the U.S. National Institute on Aging (NIA), and the research was supported in part by the Intramural Research Program, NIA, and National Institute of Health (NIH).

**Ludwigshafen Risk and Cardiovascular Health (LURIC) Study:** We thank Bernd Genser for assistance with statistical analyses.

**Medical Research Council Ely (MRC Ely) Study:** The baseline Ely Study was funded by Diabetes U.K. and the Eastern Region National Health Service Research and Development. We are most grateful to all study participants and to the staff of the St. Mary’s Street Surgery, Ely. We thank all the staff who worked on the study.

**Northern Finland Birth Cohort 1966 (NFBC 1966)**: This project was primarily funded by the MRC (project grant G0601653, PrevMetSyn/SALVE). NFBC1966 received financial support also via grants from the Academy of Finland (project grants 104781, 120315, 129269, 1114194, Center of Excellence in Complex Disease Genetics and SALVE), University Hospital Oulu, Biocenter, University of Oulu, Finland (75617), the European Commission (EURO-BLCS, Framework 5 award QLG1-CT-2000-01643), NHLBI grant 5R01HL087679-02 through the STAMPEED program (1RL1MH083268-01), NIH/NIMH (5R01MH63706:02), ENGAGE project and grant agreement HEALTH-F4-2007-201413, and the Medical Research Council, UK (G0500539, G0600705). The DNA extractions, sample quality controls, biobank up-keeping and aliquotting was performed in the National Public Health Institute, Biomedicum Helsinki, Finland and supported financially by the Academy of Finland and Biocentrum Helsinki. We thank Professor (Emeritus) Paula Rantakallio (launch of NFBC1966 and NFBC1986), and Ms Outi Tornwall and Ms Minttu Jussila (DNA biobanking). The authors would like to acknowledge the contribution of the late Academian of Science, Prof. Leena Peltonen.

**Prospective Investigation of the Vasculature in Uppsala Seniors (PIVUS) and Uppsala Longitudinal Study of Adult Men (ULSAM):** PIVUS and ULSAM studies were supported by the Swedish Research Council and Knut and Alice Wallenberg Foundation. Genotyping was performed by the SNP Technology Platform at Uppsala University, Sweden (<http://www.genotyping.se>).

**Scottish Colorectal Cancer Study (SOCCS):** This work was supported by grants from Cancer Research UK (C348/A3758 and A8896, C48/A6361), Medical Research Council (G0000657-53203) and Scottish Executive Chief Scientist's Office (K/OPR/2/2/D333, CZB/4/449), and a Centre Grant from CORE as part of the Digestive Cancer Campaign. Evropi Theodoratou is funded by Cancer Research UK Fellowship C31250/A10107.

**The Gothenburg Osteoporosis and Obesity Determinants Study (GOOD)**: The GOOD study is supported by the Swedish Research Council, the Swedish Foundation for Strategic Research, The ALF/LUA research grant in Gothenburg, the Lundberg Foundation, the Torsten and Ragnar Söderberg's Foundation, the Novo Nordisk Foundation, and the European Commission grant HEALTH-F2-2008-201865-GEFOS.

**Twins UK**: The study is supported by the Wellcome Trust (UK), NIHR Biomedical Research Centre (grant to Guys’ and St. Thomas’ Hospitals and King’s College London) and the FP-5 GenomEUtwin Project (QLG2-CT-2002-01254).

**UK Blood Services Common Control Collection (UKBS-CC)**: We acknowledge use of DNA from The UK Blood Services collection of Common Controls (UKBS-CC collection), funded by the Wellcome Trust grant 076113/C/04/Z, the Wellcome Trust/Juvenile Diabetes Research Foundation grant 061858, and the National Institute of Health Research of England. The collection was established as part of the Wellcome Trust Case-Control Consortium.

**Young Finns Study**: The Young Finns Study has been financially supported by the Academy of Finland: grants 126925, 121584, 124282, 129378, 117797, and 41071, the Social Insurance Institution of Finland, Kuopio, Tampere and Turku University Hospital Medical Funds, Juho Vainio Foundation, Paavo Nurmi Foundation, Finnish Foundation of Cardiovascular Research and Finnish Cultural Foundation, Sigrid Juselius Foundation, Tampere Tuberculosis Foundation and Emil Aaltonen Foundation.

No funding bodies had any role in study design, data collection and analysis, decision to publish, or preparation of the manuscript.
